# Supplementary material for: Improving maternal and newborn health services in Northeast Nigeria through a government-led partnership of stakeholders: a quasi-experimental study
Source: BMJ Open. 2022 Feb 1;12(2):e048877. doi: 10.1136/bmjopen-2021-048877 (PMC8808391; doi:10.1136/bmjopen-2021-048877)
Supplement: Author's manuscript [file bmjopen-2021-048877.draft_revisions.pdf]

BMJ Open is committed to open peer review. As part of this commitment we make the peer review history of every article we publish publicly available.

When an article is published we post the peer reviewers' comments and the authors' responses online. We also post the versions of the paper that were used during peer review. These are the versions that the peer review comments apply to.

The versions of the paper that follow are the versions that were submitted during the peer review process. They are not the versions of record or the final published versions. They should not be cited or distributed as the published version of this manuscript.

BMJ Open is an open access journal and the full, final, typeset and author-corrected version of record of the manuscript is available on our site with no access controls, subscription charges or pay-per-view fees (http://bmjopen.bmj.com).

If you have any questions on BMJ Open's open peer review process please email info.bmjopen@bmj.com

BMJ Open

Improving maternal and newborn health services in northeast Nigeria through a government-led partnership of stakeholders: a quasi-experimental study

Journal:	*BMJ Open*
Manuscript ID	bmjopen-2021-048877
Article Type:	Original research
Date Submitted by the Author:	09-Jan-2021

Complete List of Authors:	Willey, Barbara; London School of Hygiene and Tropical Medicine, Infectious Disease Epidemiology<br>Umar, Nasir; London School of Hygiene and Tropical Medicine Faculty of Infectious and Tropical Diseases, Disease Control<br>Beaumont, Emma; London School of Hygiene and Tropical Medicine, Infectious Disease Epidemiology<br>Allen, Elizabeth<br>Anyanti, Jennifer; Society for Family Health<br>Bello, Abubakar Bala ; PACT Nigeria<br>Bhattacharya, Antoinette Alas; London School of Hygiene and Tropical Medicine Faculty of Infectious and Tropical Diseases, Department of Disease Control<br>Exley, Josephine; London School of Hygiene and Tropical Medicine Faculty of Infectious and Tropical Diseases, Department of Disease Control<br>Makowiecka, Krystyna; London School of Hygiene and Tropical Medicine, Epidemiology and Population Health<br>Okolo, Magdalyn; Society for Family Health Nigeria<br>Sani, Rabi; PACT Nigeria<br>Schellenberg, Joanna; London School of Hygiene and Tropical Medicine, Disease Control<br>Spicer, Neil; London Sch Hyg ,<br>Usman, Umar Adamu; Data Research and Mapping Consult Limited<br>Gana, Ahmed; Gombe State Primary Health Care Development Agency<br>Shuaibu, Abdulrahman ; State Primary Health Care Development Agency Gombe State, Nigeria, GSPHCDA<br>Marchant, Tanya; London Sch Hyg , Disease Control

Keywords:	Organisation of health services < HEALTH SERVICES ADMINISTRATION & MANAGEMENT, Public health < INFECTIOUS DISEASES, NEONATOLOGY, Maternal medicine < OBSTETRICS

SCHOLARONE™
Manuscripts

I, the Submitting Author has the right to grant and does grant on behalf of all authors of the Work (as defined in the below author licence), an exclusive licence and/or a non-exclusive licence for contributions from authors who are: i) UK Crown employees; ii) where BMJ has agreed a CC-BY licence shall apply, and/or iii) in accordance with the terms applicable for US Federal Government officers or employees acting as part of their official duties; on a worldwide, perpetual, irrevocable, royalty-free basis to BMJ Publishing Group Ltd ("BMJ") its licensees and where the relevant Journal is co-owned by BMJ to the co-owners of the Journal, to publish the Work in this journal and any other BMJ products and to exploit all rights, as set out in our licence.

The Submitting Author accepts and understands that any supply made under these terms is made by BMJ to the Submitting Author unless you are acting as an employee on behalf of your employer or a postgraduate student of an affiliated institution which is paying any applicable article publishing charge ("APC") for Open Access articles. Where the Submitting Author wishes to make the Work available on an Open Access basis (and intends to pay the relevant APC), the terms of reuse of such Open Access shall be governed by a Creative Commons licence – details of these licences and which Creative Commons licence will apply to this Work are set out in our licence referred to above.

Other than as permitted in any relevant BMJ Author's Self Archiving Policies, I confirm this Work has not been accepted for publication elsewhere, is not being considered for publication elsewhere and does not duplicate material already published. I confirm all authors consent to publication of this Work and authorise the granting of this licence.

For peer review only

Improving maternal and newborn health services in northeast Nigeria through a government-led partnership of stakeholders: a quasi-experimental study

Barbara Willey[1], Nasir Umar[1], Emma Beaumont[1], Elizabeth Allen[1], Jennifer Anyanti[4], Abubakar Bala Bello[3], Antoinette Bhattacharya[1], Josephine Exley[1], Krystyna Makowiecka[1], Magdalene Okolo[4], Rabi Sani[3], Joanna Schellenberg[1], Neil Spicer[1], Umar Adamu Usman[2], Ahmed Mohammed Gana[7], Abdulrahman Shuaibu[8], Tanya Marchant[1]

Affiliations

[1] London School of Hygiene and Tropical Medicine, London, UK

[2] Data Research and Mapping Consult Limited, Abuja, Nigeria

[3] Pact, Abuja, Nigeria

[4] Society for Family Health, Abuja, Nigeria

[7] Office of the Honourable Commissioner for Health (and former Executive Secretary GSPCDA), Gombe State Ministry, Gombe, Nigeria

[8] Office of the Executive Secretary, Gombe State Primary Health Care Development Agency, Gombe, Nigeria

For peer review only - http://bmjopen.bmj.com/site/about/guidelines.xhtml

Abstract

Objectives

This study aimed to quantify change in the coverage, quality and equity of essential maternal and newborn health care interventions in Gombe state, northeast Nigeria, following a four year, government-led, maternal and newborn health intervention.

Design

Quasi-experimental plausibility study. Repeat cross-sectional household and linked health facility surveys were implemented in intervention and comparison areas.

Setting

Gombe State, northeast Nigeria.

Participants

Each household survey included a sample of 1,000 women aged 13-49 years with a live birth in the previous twelve months. Health facility surveys comprised a readiness assessment and birth attendant interview.

Interventions

Between 2016-2019 a complex package of evidence-based interventions was implemented to increase access, use and quality of maternal and newborn health care, spanning the six WHO health system building blocks.

Outcome measures

Eighteen indicators of maternal and newborn health care.

Results

Between 2016 and 2019, the coverage of all indicators improved in intervention areas, with the exception of post-natal and post-partum contacts, which remained below fifteen percent. Greater improvements were observed in intervention than comparison areas for eight indicators, including coverage of at least one antenatal visit (71% (95% CI 62-68) to 88% (95% CI 82-93)), at least four antenatal visits (46% (95% CI 39-53) to 69% (95% CI 60-75)), facility birth (48% (95% CI 37-59) to 64% (95% CI 54-73)), administration of uterotonics (44% (95% CI 34-54) to 59% (95% CI 50-67)), delayed newborn bathing (44% (95% CI 36-52) to 62% (95% CI 52-71)), and clean cord care (42% (95% CI 34-49) to 73% (95% CI 66-79)). Wide-spread inequities persisted however; only at least one antenatal visit saw pro-poor improvement.

Conclusions

This intervention achieved improvements in life-saving behaviours for mothers and newborns, demonstrating that multi-partner action, coordinated through government leadership, can shift the needle in the right direction, even in resource constrained settings.

For peer review only - http://bmjopen.bmj.com/site/about/guidelines.xhtml

Strengths and limitations of the study

- This was a large, multi-year study to comprehensively measure improvements in access, use, quality and equity of maternal and newborn health care in a setting with a high mortality burden.
- Standardised tools were applied each year to collect both population level and facility level evidence.
- The study identified improvements across a broad range of maternal and newborn indicators but also revealed that this government-led intervention did not improve postnatal care and did not redress pre-existing socio-economic inequalities.
- The selection of intervention and comparison areas was pragmatic, following government implementation plans, and baseline characteristics between areas were not balanced.
- Despite adjustment in multi-variate models, residual confounding by area cannot be discounted.

For peer review only

For peer review only - http://bmjopen.bmj.com/site/about/guidelines.xhtml

INTRODUCTION

Interventions to reduce maternal and newborn morbidity and mortality are available (1-6), however, implementation gaps create a mismatch between the efficacy and effectiveness of interventions at a population level (7-9). Despite substantial progress in some regions during the era of the Millennium Development Goals (10), maternal and neonatal mortality remain unacceptably high, especially in low- and middle-income countries (11-13). The era of Universal Health Coverage highlights the need for a focus on quality of care and reducing inequalities alongside increasing access to care (14-19).

Challenges remain in how to equitably scale and package efficacious interventions and integrate them within existing health systems (20). Examining maternal, newborn, and child health coverage data from 36 sub-Saharan African countries, Wehrmeister and colleagues concluded that countries had narrowed equity gaps since 1995, although wealth-related inequalities remain highest in West Africa. (21). In their study of 83 low- and middle-income countries, Barros and colleagues emphasised the need for implementation of pro-poor strategies if the most vulnerable are to benefit from available health care (22). Part of the solution to these challenges may be robust governance and government-led initiatives that focus on universal and equitable access, (23, 24).

Here we report the effect of the Gombe Partnership for Maternal and Newborn Health, a government-led intervention implemented between 2016 and 2019 in north-eastern Nigeria and designed to coordinate multiple actors toward the goal of equitably improving high quality maternal and newborn health services. An intended outcome of the partnership was to inform scale up and share learning across neighbouring states and regions. Implementation used an adaptive management approach and partners met regularly to examine monitoring data and amend implementation as necessary. In this paper, we examine the effect of this intervention on the coverage and quality of essential maternal and newborn health care interventions after four years of implementation, and assess whether any improvements were equitable across socio-economic groups.

For peer review only - http://bmjopen.bmj.com/site/about/guidelines.xhtml

METHODS

Setting and Intervention implementation

This study was conducted in Gombe State, a predominately rural (80%) and sparsely-populated state in north-eastern Nigeria (25), where the burden of maternal and neonatal mortality is higher than the national average and stood at 1,549 maternal deaths per 100,000 live births in 2015 and 33 neonatal deaths per 1,000 live births in 2017 (26, 27). In 2016, when this study started, 29% of women gave birth in a health facility (25), principally a primary health centre (PHC) (28, 29). Primary health care services are predominately delivered by the Gombe State Primary Health Care Development Agency (30). In 2016, 460 PHCs in the state provided antenatal care (ANC), birth and intrapartum services (28), mainly delivered by community health extension workers (CHEWs), junior CHEWs and community health officers, but very few nurses, midwives or doctors (31).

From 2016-2019, the government-led a maternal and newborn health partnership to improve access, use and quality of maternal and newborn health services. Within this partnership, non-governmental organisations (NGOs) implemented a package of evidence-based interventions that spanned the six WHO health system building blocks (32) (figure 1). The government, NGOs, partners and the funder met six-monthly to review monitoring data, trouble-shoot implementation challenges, course-correct, and reinvigorate communal purpose towards a shared goal. Certain interventions were delivered on a state-wide basis, such as advocacy activities, but the majority were implemented in an intervention area, with a view of scaling-up to the entire state. The intervention area was defined as 57 sub-district level wards (half of the state's 114 wards), purposively identified by government. Community-based demand generation activities were implemented in these wards, and one centrally located PHC within each ward was chosen to implement the activities designed to improve health service quality: the rationale being that it was preferable to have one well-functioning PHC per ward, rather than a larger number of less well-functioning facilities. Residents of the state's remaining 57 wards (comparison area) continued to receive their usual care, with the exception of state-wide components (figure 1).

For peer review only

Figure 1: Intervention components by Health System Strengthening Building Block

**WHO HSS building block**	**Service Delivery**	**Health Workforce**	**Information**	**Products and technologies**	**Financing**	**Leadership Governance**
Implemented in intervention area only	Quality improvement teams in PHCs<br>VHW links with facilities<br>Forum of mothers in law<br>Forum of males<br>Mothers groups	Task shifting and training CHEWs<br>Training and deployment of VHWs<br>Financial incentives for VHWs		Enhancing supply chains to PHCs		Strengthening Ward Development Committees<br>Organizational development
Implemented state-wide	Emergency transport Scheme		HMIS strengthening<br>MPDSR information package and support to committee<br>Score cards<br>Mass media events		Leveraging the State Level Accountability Mechanism to advocate for appropriate budget release for MNH	

Health System strengthening (HSS), Primary Health Centre (PHC), Community Health Extension Worker (CHEW), Village Health Worker (VHW), Health Management Information System (HMIS), Maternal Perinatal Death Surveillance and Response (MPDSR), Maternal and Newborn Health (MNH).

For peer review only

For peer review only - http://bmjopen.bmj.com/site/about/guidelines.xhtml

Study design

We used a quasi-experimental plausibility study design (33) to explore the association between the intervention and indicators of use and quality of maternal and newborn health services. Repeat cross-sectional household and linked health facility surveys were undertaken. Survey methods were replicated each time. Data collection tools were informed by existing large scale survey tools such as the Demographic and Health Surveys (34) and Service Availability and Readiness Assessment Surveys (35). We conducted pre-testing of questions and pilot testing of survey protocols, including translation and back-translation of survey questions between English and Hausa.

We conducted four annual household surveys during July/August 2016-2019. A random sample of 80 clusters was selected: 40 each from intervention and comparison wards. Clusters were segmented enumeration areas as defined by the National Population Commission. Within each cluster, all households were visited. Following consent procedures, a modular household questionnaire was conducted in Hausa by trained interviewers. During each survey, we aimed to survey a total of 6,000 households across the 80 clusters. This was expected to result in interviews with 1,000 women with a live birth in the previous 12 months (i.e. 500 each in intervention and comparison). This sample size was sufficient to measure as statistically significant (95% probability), and with 80% power, changes of 15 percentage points in a range of coverage indicators.

During the same period, we conducted seven facility and birth attendant surveys at six-monthly intervals. Four surveys were done concurrently with the household surveys (figure 2). A facility readiness assessment was carried out plus an interview was conducted with the available birth attendant who had attended the most recent delivery recorded in the maternity register. All 57 intervention PHCs were surveyed plus one PHC selected at random from each of the 40 comparison wards sampled during household surveys. This sample of 97 PHCs was sufficient to measure as statistically significant (95% probability), and with 80% power, changes of 30 percentage points in a range of indicators of availability of essential commodities and medicines.

All data were collected using hand-held digital devices and synchronized with a supervisor laptop each day. Automated summary reports were produced to identify and address any internal inconsistencies.

Statistical analysis of effectiveness and equity

Eighteen indicators measuring the coverage of life-saving commodities and behaviours were selected *a priori* for their known association with maternal and newborn health outcomes (box 1). Changes over the four-year study period were compared between intervention and comparison areas. Analyses were at the individual level for household survey indicators and at the health facility level for facility and birth attendant indicators. Data were analysed in Stata 15 (StataCorp, 2017, College Station, TX).

Household and health facility characteristics were summarised using appropriate summary statistics. We used survey commands (*svy*) to account for clustering. We identified variables which differed significantly between intervention and comparison areas in 2016 using a design-based F test and included their cluster-level means in regression models. Percentage point differences and 95% CIs in indicator coverage between 2016 and 2019 were calculated.

Effectiveness of the programme on pre-specified indicators was estimated through logistic regression models. A likelihood ratio test comparing models with time as a continuous variable to

For peer review only - http://bmjopen.bmj.com/site/about/guidelines.xhtml

one where each time point was included separately, determined whether time was included as a continuous or categorical variable. Models included fixed effects for area (intervention vs comparison), time (at all time points) and the interaction between area and time, to describe any additional effect in 2019 in the intervention areas compared to 2016 in the comparison areas. Cluster level means of variables which differed between intervention and comparison areas in 2016, identified using a design-based F-test, were included in all models. We included a random effect for cluster to account for clustering in logistic regression models of household survey indicators.

To examine equity, principal components analysis was used to generate an index of household wealth, based on asset ownership (36). Using this, households were categorised into quintiles from poorest to least poor. Where there was evidence of greater improvement in the intervention areas compared to the comparison areas, we examined the difference of change over time by household socioeconomic quintile in the intervention areas. We tested for interaction between time and socioeconomic status quintile.

Ethics

This research was conducted with approval from: The Nigerian National Health Research Ethics Committee (NHREC/01/01/2007); the State Ministry of Health Gombe State (ADM/S/658/Vol. II/66); and the London School of Hygiene & Tropical Medicine (12181). The free and informed consent of all participants was obtained.

Patient and public involvement

Patients and the public were not involved in the design, conduct, reporting or dissemination plans of our research.

For peer review only - http://bmjopen.bmj.com/site/about/guidelines.xhtml

Figure 2: Study timeline

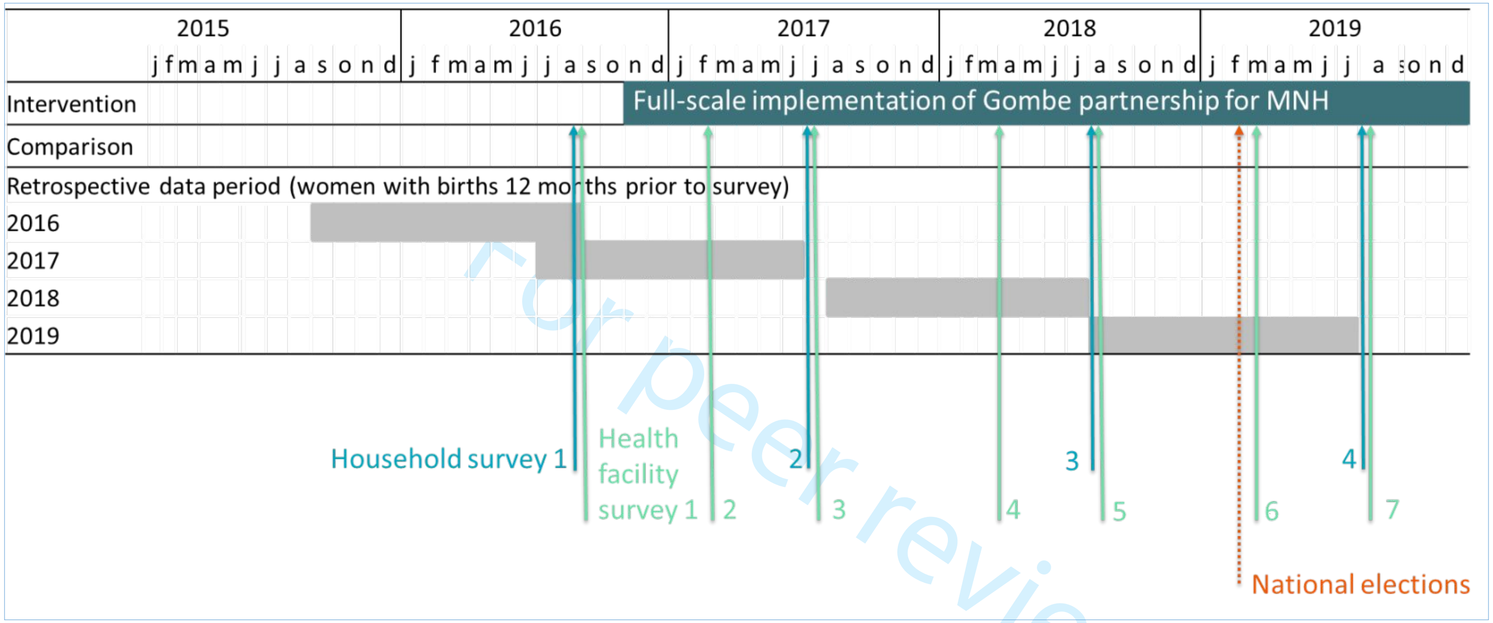

Maternal and Newborn Health (MNH)

For peer review only - http://bmjopen.bmj.com/site/about/guidelines.xhtml

Box 1: Indicator definitions

Indicator name	Numerator	Denominator
Antenatal care period		
Women made at least one ANC visit	Number of women aged 15-49 years with a birth during 12 months before survey who made at least one ANC visit	Women aged 15-49 years with a birth during 12 months before survey
Women made at least four ANC visits	Number of women aged 15-49 years with a birth during 12 months before survey who made at least four ANC visits	Women aged 15-49 years with a birth during 12 months before survey
Women received basic ANC quality	Number of women aged 15-49 years with a birth during 12 months before survey attending ANC who received all three of: urine test, blood test, and blood pressure measurement	Women aged 15-49 years with a birth during 12 months before survey
Women received syphilis test results	Number of women aged 15-49 years with a birth during 12 months before survey who received a test result for syphilis	Women aged 15-49 years with a birth during 12 months before survey
Facilities had syphilis treatment available	Number of primary health facilities which had benzathine penicillin for syphilis treatment in stock	Primary health facilities surveyed
Intrapartum care period		
Facility delivery	Number of women aged 15-49 years with a birth during 12 months before survey who gave birth in a health facility	Women aged 15-49 years with a birth during 12 months before survey
Uterotonics	Number of women aged 15-49 years with a birth during 12 months before survey who received prophylactic uterotonics, includes facility and home births	Women aged 15-49 years with a birth during 12 months before survey
Facilities had pre-eclampsia treatment available	Number of primary health facilities which had magnesium sulphate available for treatment of pre-eclampsia	Primary health facilities surveyed
Birth attendant was able to correctly manage pre-eclampsia	Number of birth attendants who were able to correctly manage pre-eclampsia, as assessed by clinical vignette	Birth attendants interviewed
Immediate newborn care period		
Facilities had newborn resuscitation equipment available	Number of primary health facilities which had a bag and mask for newborn resuscitation (size 0 and size 1)	Primary health facilities surveyed
Birth attendant was able to correctly manage newborn resuscitation	Number of birth attendants who were able to correctly manage neonatal resuscitation, as assessed by simulation	Birth attendants interviewed
Newborn bathing was delayed until at least 24 hours after birth	Number of newborns born to women aged 15-49 years with a birth during 12 months before survey who had delayed bathing for the first 24 hours of life	Women aged 15-49 years with a birth during 12 months before survey
Newborn received clean cord care	Number of newborns women aged 15-49 years with a birth during 12 months before survey who received clean cord care, defined as cutting cord with clean blade or scissors, administering chlorhexidine to the newborn	Women aged 15-49 years with a birth during 12 months before survey

For peer review only - http://bmjopen.bmj.com/site/about/guidelines.xhtml

Indicator name	Numerator	Denominator
	cord, and not administering anything harmful to the newborn cord	
Newborn was breastfed within 1 hour of birth	Number of newborns born to women aged 15-49 years with a birth during 12 months before survey for which breastfeeding was initiated within 1 hour of birth	Women aged 15-49 years with a birth during 12 months before survey
Post-partum and post-natal care period		
Women received a post-partum check within 2 days of delivery	Number of women aged 15-49 years with a birth during 12 months before survey who had a post-partum check-up within 2 days of delivery	Women aged 15-49 years with a birth during 12 months before survey
Newborns received a post-natal check within 2 days of birth	Number of newborns born to women aged 15-49 years with a birth during 12 months before survey who had a post-natal check-up within 2 days of birth	Women aged 15-49 years with a birth during 12 months before survey
Newborns with suspected sepsis treated with antibiotics	Number of newborns aged 0-60 days with suspected sepsis who received antibiotics	Newborns aged 0-60 days with suspected sepsis
Facilities had newborn sepsis treatment available	Number of primary health facilities with amoxicillin and gentamicin available	Primary health facilities surveyed

Note: data were collected for women aged 13–49 years. However, no women <15 years of age had a live birth in the 12 months before the survey. Antenatal care (ANC).

For peer review only

For peer review only - http://bmjopen.bmj.com/site/about/guidelines.xhtml

RESULTS

Sample description

Table 1 shows the number of households, resident women, and facilities surveyed. High response rates were seen throughout. Table 2 presents baseline characteristics for the 1,011 women interviewed with a recent live birth. Women had a mean age of 26 years (SD 0.4) and parity of four births (SD 0.2); over one quarter reported more than six births. Marriage was near universal (>94%) and 82% of women were Muslim. Mean year of schooling was 4.3 (SD 0.6) in intervention and 2.6 years (SD 0.5) in comparison (p=0.06). Although the majority reported no formal education, this was substantially higher in comparison areas (72% vs. 55%, p=0.05).

Table 1: Interviews conducted, by survey and year

	**2016**	**2017**	**2018**	**2019**
Number of clusters	80	80	80	80
Households				
Target number of households to be surveyed	6000	6000	6000	6000
Number of households surveyed	5747	5762	5925	5616
Response rate	96%	96%	99%	94%
Resident women				
Number of resident women aged 15-49 years	8556	8395	10177	10080
Number of women interviewed	8453	8270	10150	9959
Response rate	99%	98%	99%	99%
Number of resident women with live birth in 12 months preceding survey	1011	932	991	871
Primary health facilities				
Target number of primary health facilities to be surveyed	97	97	97	97
Number of primary health facilities surveyed	94	94	97	97
Response rate	97%	97%	100%	100%
Number of birth attendants interviewed (one per facility)	92	91	93	95
Response rate	95%	94%	96%	98%

Note: The survey sampled resident women aged 13-49 years, however, no births in the last twelve months were recorded among women aged 13-14 years and as such the sample of women with a recent birth is aged 15-49 years.

For peer review only

Table 2: Baseline characteristics: women with recent birth (2016 survey)

	Intervention		Comparison		p-value**
	N=519	% (95% CI)	N=492	% (95% CI)	
Woman's age category					
15-19 years	54	10 (8-14)	60	12 (9-16)	0.35
20-24 years	163	32 (27-37)	128	26 (23-30)	
25-29 years	146	28 (25-32)	128	26 (22-31)	
30-34 years	87	17 (14-20)	94	19 (16-23)	
35-39 years	48	9 (7-12)	56	11 (9-14)	
40-44 years	15	3 (2-5)	21	4 (3-7)	
45-49 years	3	0.6 (0.2-2)	4	0.8 (0.3-2)	
Parity					
1 birth	111	21 (18-26)	79	16 (13-20)	0.07
2 births	76	15 (11-19)	83	17 (14-20)	
3-5 births	205	39 (35-44)	178	37 (32-41)	
≥ 6 births	127	25 (20-30)	152	31 (27-35)	
Marital status					
Currently married	488	94 (89-97)	472	96 (93-98)	0.35
Not married	31	6 (3-10)	20	4 (2-7)	
Religion					
Christianity	92	18 (10-31)	71	14 (8-25)	0.62
Islam	426	82 (69-90)	421	86 (75-92)	
Education level completed					
None	283	55 (42-67)	353	72 (60-81)	0.05
1-7 years (primary)	87	17 (11-24)	56	11 (7-19)	
≥ 8 years (secondary)	149	29 (20-39)	83	17 (11-26)	
*Household socioeconomic status, by quintile**					
Quintile 1 (poorest)	102	20 (14-27)	113	23 (15-33)	0.44
Quintile 2	96	19 (14-24)	111	23 (17-29)	
Quintile 3	114	22 (17-27)	96	20 (15-24)	
Quintile 4	94	18 (14-24)	100	20 (15-27)	
Quintile 5 (richest)	113	22 (14-33)	72	15 (9-23)	

* Socioeconomic asset index: wall material not thatch or mud, floor material not earth or sand, clean source of drinking water, roof material iron, tiles or cement, flush toilet or latrine, electricity supply to home, ownership of fridge, TV, radio, bike, kerosene lamp, wrist watch, motorcycle, generator, fan. ** p-value from design-based F test

For peer review only - http://bmjopen.bmj.com/site/about/guidelines.xhtml

Table 3 shows that in 2016, almost all sampled facilities provided ANC and delivery services, and at least 70% of facilities provided postnatal care. Only a small minority of facilities had a trained midwife available 24 hours a day. In total, a third of intervention PHCs, and a quarter of comparison PHCs provided all basic emergency obstetric and newborn care (BEmONC) signal functions (excluding assisted vaginal delivery) in the previous three months (p=0.58). Facilities had low volumes of deliveries (<500 births per year) (40), with each intervention PHC managing a mean of 288 births per year versus 192 in comparison PHCs.

Table 3: Baseline characteristics: health facilities (2016 survey)

	Intervention		Comparison		p-value *
	N=55	(%, 95% CI)	N=39	(%, 95% CI)	
Infrastructure					
Clean source of running water	34	62 (48-74)	17	44 (29-60)	0.09
Electricity connection	38	69 (56-80)	24	62 (45-76)	0.45
Opening hours					
7 days a week	44	80 (67-89)	25	68 (51-81)	0.18
Services offered					
ANC services available	55	100	37	95 (81-99)	0.09
Delivery services available	55	100	34	87 (72-95)	0.01
Post-natal care services available	42	76 (63-86)	27	69 (53-82)	0.45
24hour delivery services available 7 days a week	44	83 (70-91)	27	82 (65-92)	0.08
Trained midwife available 24 hours a day 7 days a week	14	15 (9-24)	12	13 (7-21)	0.57
Provided all BEmONC signal functions (excluding assisted vaginal delivery) in previous 3 months	17	31 (20-45)	10	26 (14-42)	0.58

Antenatal care (ANC), Basic emergency obstetric and newborn care (BEmONC). * p-value from design-based F test

For peer review only

Change over time in intervention and comparison areas

Table 4 shows prevalence of 18 maternal and newborn health indicators between 2016 and 2019 in intervention and comparison areas for women with a recent live birth, PHCs, and surveyed birth attendants. With the exception of post-partum and post-natal health checks, which did not improve, 16 indicators improved over time in the intervention area (table 4).

Table 4 shows that seven indicators improved in the intervention areas only, while nine also improved in comparison areas. For eight indicators, greater changes over time were observed in intervention areas above any increases seen in comparison areas. The proportion of women attending ANC visits and giving birth in a facility increased to 88% for ANC1, 69% for ANC4 and 64% for facility birth. These represented improvements up to nine percentage points above increases seen in comparison areas (p=0.006 and p=0.003, respectively for ANC1 and ANC4, and p<0.001 for facility birth). For the intrapartum period, we saw an increase in the administration of prophylactic uterotonics among all women reaching 59%, a four percentage point improvement above changes in the comparison areas (p=0.008). Availability of treatment for pre-eclampsia increased by 32 percentage points above changes in the comparison areas (p=0.021). Adoption of life-saving behaviours for immediate newborn care also increased, with improvements up to 10 percentage points higher than changes seen in comparison areas (p=0.034 for delayed bathing of the newborn, and p<0.001 for clean cord care, respectively). Availability of newborn bag and mask for resuscitation increased by 36 percentage points above changes in the comparison areas (p=0.001). Similar improvements were observed in intervention and comparison areas for three indicators: women receiving syphilis test results (p=0.758); birth attendants' capability to manage pre-eclampsia appropriately (p=0.971), and immediate breastfeeding of the newborn (p=0.524) (table 4).

For peer review only

For peer review only - http://bmjopen.bmj.com/site/about/guidelines.xhtml

Table 4: Indicators over time by intervention and comparison area

Indicator	Area	2016			2017			2018			2019			Percentage point difference 2019-2016 (95% CI) p-value*	Linear trend p-value**	p-value interaction time and area ***
		N	n	% (CI)	N	n	% (CI)	N	n	% (CI)	N	n	% (CI)			
**Antenatal care period**																
Women made at least one ANC visit	Intervention	519	366	71 (62-78)	494	350	71 (64-77)	511	435	85 (79-90)	458	405	88 (82-93)	17 (11-25) <0.001	<0.001	0.006
	Comparison	492	279	57 (47-66)	438	239	55 (46, 63)	480	292	61 (51-70)	413	298	72 (61-81)	15 (6-25) 0.002	0.014	
Women made at least four ANC visits	Intervention	519	239	46 (39-53)	494	230	46 (39-53)	511	326	64 (56-71)	511	315	69 (60-76)	23 (14-32) <0.001	0.002	0.003
	Comparison	493	145	29 (23-37)	438	131	30 (23-37)	480	164	34 (25-45)	480	179	43 (34-53)	14 (6-22) <0.001	0.458	
Women received basic ANC quality	Intervention	519	303	58 (50-66)	494	261	53 (44-62)	511	312	61 (52-69)	458	305	66 (58-74)	8 (1-18) 0.008	0.002	0.599
	Comparison	492	216	44 (35-53)	438	160	37 (29-45)	480	183	38 (30-47)	413	220	53 (42, 64)	9 (-0.2-19) 0.056	NA	
Women received syphilis test results	Intervention	519	61	12 (9-16)	494	86	17 (12-24)	511	166	32 (26-40)	458	149	33 (27-38)	21 (14-27) <0.001	<0.001	0.758
	Comparison	492	39	8 (6-11)	438	55	13 (8-20)	480	96	20 (14-27)	413	88	21 (15-30)	13 (5-21) 0.002	0.008	
Facilities PHC had syphilis treatment available	Intervention	55	5	9 (4-20)	55	29	53 (39-66)	57	37	65 (52-76)	57	21	37 (25-50)	28 (12-44) <0.001	<0.001	0.061
	Comparison	39	3	8 (2-22)	39	7	18 (9-33)	40	6	15 (7-30)	41	5	12 (5-26)	4 (-8-17) 0.467	NA	
**Intrapartum care period**																
Women gave birth in a facility	Intervention	519	248	48 (37-59)	494	217	44 (33-55)	511	313	61 (53-69)	458	294	64 (54-73)	16 (7-26) 0.002	<0.001	<0.001
	Comparison	492	128	26 (18-35)	436	154	35 (27-45)	480	147	31 (23-39)	412	145	35 (26-46)	9 (0.5-18) 0.039	0.078	
Women received uterotonic	Intervention	509	223	44 (34-54)	473	186	39 (30-49)	505	264	52 (45-60)	435	256	59 (50-67)	15 (6-24) 0.001	0.002	0.008
	Comparison	487	133	27 (20-37)	416	127	31 (23-40)	475	132	28 (21-35)	372	141	38 (29-48)	11 (3-19) 0.010	0.527	

For peer review only

PHC had pre-eclampsia treatment available	Intervention	55	11	20 (11-33)	55	35	64 (51-75)	57	56	98 (88-100)	57	53	93 (82-97)	73 (60-86) <0.001	<0.001	0.021
	Comparison	39	5	13 (5-28)	39	7	18 (9-33)	40	23	58 (42-72)	41	22	54 (38-68)	41 (24-58) <0.001	0.062	
Birth attendant was able to correctly manage pre-eclampsia	Intervention	55	16	29 (18-43)	55	23	42 (29-55)	57	46	81 (68-89)	57	50	88 (76-94)	59 (42-75) <0.001	0.023	0.971
	Comparison	37	10	27 (15-44)	36	11	31 (18-48)	36	19	53 (36-69)	38	27	71 (55-83)	44 (24-64) <0.001	0.005	
**Immediate newborn care period**																
Facilities had newborn resuscitation equipment available	Intervention	55	35	64 (50-75)	55	45	82 (69-90)	57	51	89 (78-95)	57	54	95 (85-98)	31 (17-46) <0.001	<0.001	0.001
	Comparison	39	17	44 (29-60)	39	12	31 (18-47)	40	8	20 (10-35)	41	16	39 (25-55)	-5 (-18-9) 0.509	NA	
Birth attendant was able to correctly manage newborn resuscitation	Intervention	55	6	11 (5-23)	55	12	22 (13-35)	57	27	47 (35-60)	57	27	47 (35-60)	36 (19-54) <0.001	0.038	0.775
	Comparison	37	5	14 (6-29)	36	5	14 (6-30)	36	8	22 (11-39)	38	9	24 (13-40)	10 (-8-29) 0.276	NA	
Newborn bathing was delayed until at least 24 hours after birth	Intervention	519	226	44 (36-52)	492	224	46 (37-54)	511	305	60 (50-69)	458	285	62 (52-71)	18 (8-30) 0.002	0.012	0.034
	Comparison	492	161	33 (26-40)	435	136	31 (24-39)	480	188	39 (30-49)	412	169	41 (32-51)	8 (-1-18) 0.090	NA	
Newborn received clean cord care	Intervention	519	216	42 (34-49)	494	212	43 (36-50)	511	331	65 (57-72)	458	335	73 (66-79)	31 (21-42) <0.001	<0.001	<0.001
	Comparison	492	140	28 (23-35)	438	134	31 (24-38)	480	159	33 (26-41)	413	208	50 (41-59)	22 (13-31) <0.001	0.028	
Newborn was breastfed within 1 hour of birth	Intervention	511	270	53 (46-60)	489	283	58 (49-66)	509	531	69 (62-75)	454	304	67 (59-74)	14 (3-25) 0.016	0.028	0.524
	Comparison	483	215	45 (39-50)	430	207	48 (40-56)	474	262	55 (49-62)	409	249	61 (51-70)	16 (7-26) 0.002	0.586	
**Post-partum and post-natal care period**																
Women received a post-partum check within 2 days of delivery	Intervention	519	56	11 (7-17)	492	39	8 (5-12)	507	59	12 (8-17)	457	60	13 (9-19)	2 (-4-9) 0.451	NA	0.018
	Comparison	492	24	5 (3-9)	434	23	5 (3-8)	480	11	2 (1-5)	413	34	8 (5-13)	3 (-1-8) 0.002	0.002	
Newborns received a post-natal check within 2 days of birth	Intervention	519	28	5 (3-9)	491	27	5 (3-10)	510	32	6 (4-10)	458	31	7 (4-11)	2 (-2- 5) 0.391	NA	0.059
	Comparison	492	18	4 (2-7)	435	10	2 (1-4)	479	5	1 (0.3-3)	412	17	4 (2-10)	0 (-4-4) 0.825	NA	

For peer review only - http://bmjopen.bmj.com/site/about/guidelines.xhtml

Newborns with suspected sepsis treated with antibiotics	Intervention	43	15	35<br>(22-50)	33	13	39<br>(23-58)	25	9	36<br>(15-64)	15	11	73<br>(44-91)	38 (12-65)<br>0.006	0.198	0.273
	Comparison	29	13	45<br>(28-62)	23	9	39<br>(24-57)	20	4	20<br>(9-38)	21	10	48<br>(26-70)	3 (-27, 33)<br>0.852	NA	
Facilities had newborn sepsis treatment available	Intervention	55	20	36<br>(25-50)	55	38	69<br>(56-80)	57	36	63<br>(50-75)	57	38	67<br>(53-78)	31 (10-50)<br>0.004	<0.001	0.054
	Comparison	39	12	31<br>(18-47)	39	15	38<br>(24-55)	40	9	23<br>(12-38)	41	11	27<br>(15-43)	-4 (-22-14)<br>0.663	NA	

[*] P-value based on t-test. [**] Test for linear trend based on likelihood ratio test. [***] P-value for interaction between area (intervention) and survey year (2019). Models of household survey indicators adjusted for maternal education. Denominators vary due to missing data.

For peer review only - http://bmjopen.bmj.com/site/about/guidelines.xhtml

Change in coverage 2016-19 by household wealth quintile

Six indicators from the household survey data demonstrated population level coverage improvement over time and were included in the equity analysis (Figure 3). Five of these were inequitable at baseline, with lower coverage among the poorest households. By 2019, although the change over time in coverage was even across all quintiles, baseline patterns of inequity persisted for three indicators: women attending at least four ANC visits (p=0.095), administration of uterotonics (p=0.118), and delayed bathing of the newborn (p=0.671). The improvement over time was inequitable for two indicators, with women in the poorest households benefiting the least: clean cord care (p=0.020) and facility birth (p=0.005). By contrast, improvement over time for at least one ANC was pro-poor (p=0.055), with coverage improving by 17 percentage points among the poorest households (from 58% to 75%), and by five percentage points to 91% among women in the least-poor households.

For peer review only

For peer review only - http://bmjopen.bmj.com/site/about/guidelines.xhtml

Figure 3: Equiplot of change in coverage 2016-19 by household wealth quintile

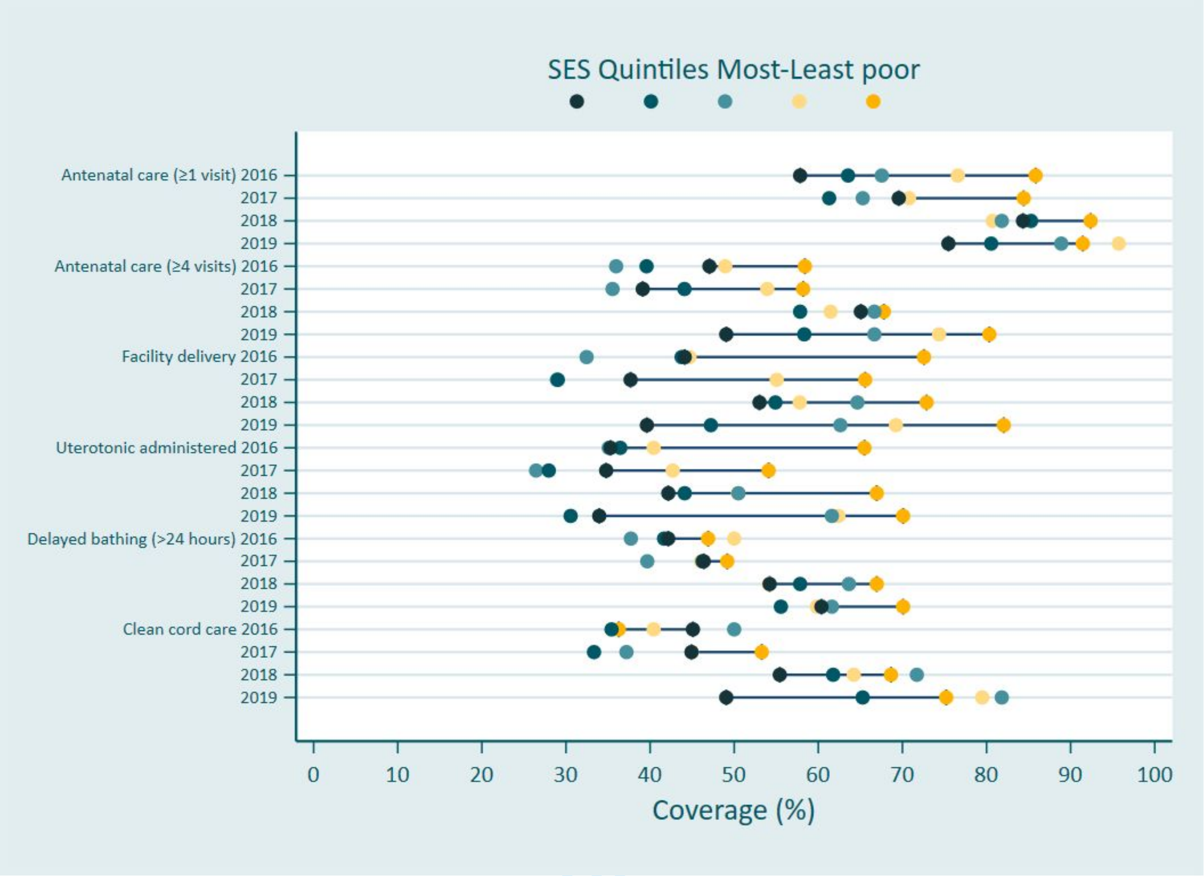

For peer review only - http://bmjopen.bmj.com/site/about/guidelines.xhtml

DISCUSSION

Over a four-year period in this rural and resource-constrained setting, this government-led maternal and newborn health partnership was able to improve several high impact indicators over and above any background changes observed in comparison areas. However, there was a marked lack of improvement in timely post-natal or post-partum care, coverage of which remained below fifteen percent. Additionally, despite improvements in coverage indicators, socio-economic inequalities in the use of maternal and newborn health services generally persisted.

Multiple improvement efforts were implemented simultaneously by the partnership. For example, increases in ANC visits and facility births were supported by more than one implementing partner. This reinforcement enabled the utilization coverage for these services to improve, even when historical data demonstrated little change during previous initiatives (38, 39). Changes to quality of care were mixed. We saw substantial increases in input quality where implementing partner actions directly strengthened pre-existing systems, for example where NGOs strengthened and managed existing supply chains for medicines and commodities and returned management to the government in a phased manner. However, this success was not replicated with process quality indicators. There was no excess improvement in process indicators of quality ANC, such as measuring blood pressure and taking a urine and blood sample during an ANC visit. Staffing and workload were especially challenging and it was not uncommon for one staff member to be attending to multiple women and babies (43). The challenge of changing health worker behaviour is well-established in the literature (40, 41). Challenges in providing complete and timely care have been noted in other low and middle income settings (44).

Coverage of timely post-natal and post-partum care for both home and facility births remained persistently low. This challenge is not unique to Gombe State, with very low coverage shown in other states of Nigeria (25), and the sub-Saharan African region more widely (42). Commonly reported barriers were present, for example sub-optimal demand generation, inadequate staffing and workload challenges, and multiple socio-cultural barriers (45, 46). However, the power of the partnership to address other apparently intractable problems was not harnessed in relation to timely post-natal and post-partum care visits, and actors did not conceive successful mechanisms that could address the problem in their setting. The role of community-based ward development committees, whose members include fathers, husbands and community leaders, had an important role in demand-generation, advocacy and accountability in this setting. NGO partners aimed to leverage the power and influence of ward committees for postnatal health, but these were enacted towards the end of our study period. Nonetheless, we suggest that such engagement is important for shifting norms and expectations where cultural barriers may restrict new mothers from returning to facilities soon after birth or accepting non-familial visitors during the immediate post-partum period (45, 46).

The Gombe Partnership for Maternal and Newborn Health achieved greater success than other recent NGO driven attempts and this study suggests the importance of the nature of the partnership and the mechanisms of implementation. The partnership was government-led with priorities and implementation guided by policy priorities (47, 48). This engendered political will. Further, good leadership, governance and accountability facilitated successful implementation despite a change in government during the 2019 general elections, which fell six months before the final data collection period of this study. The importance of political engagement and leadership has been highlighted by many, including the extensive work of Gilson and colleagues (49). From this foundation of cooperation, the intervention components and implementation plan were built to achieve a common goal. From the inception, a plan for sustainability and transition from NGO implementation

For peer review only - http://bmjopen.bmj.com/site/about/guidelines.xhtml

and donor-financing was in place (24). A substantial focus on community participation and accountability was also included, principally through the role of ward development committees.

Implementation was pragmatic and a strong emphasis was placed on adoption into the health system (20). The government and partners met six monthly to examine monitoring and evaluation data, assess progress, highlight challenges and plan for improvements. A recent systematic review has reported that many of these approaches, including a process of data-based feedback and adaptation have been important in the vertical scale-up and institutionalisation of public health interventions A recent systematic review has reported that many of these approaches, including a process of data-based feedback and adaptation have been important in the vertical scale-up and institutionalisation of public health interventions (50).

Strengths and limitations

This was a large and adequately-powered study which drew on multiple data sources and time points, and consistently applied globally recommended measurement standards. However, there were limitations. Selection of intervention areas by government was pragmatic and baseline characteristics between areas were not balanced. Despite adjustment in multi-variate models and an analysis strategy that adjusted for baseline starting points, some residual confounding by area may remain, potentially overestimating effects of the intervention. The selection of intervention areas and the inability to build in buffer zones also resulted at times in close geographic boundaries between comparison and intervention areas. This may have enabled spill-over of effects created by women seeking care and utilising services from a facility that did not correspond to the intervention status of the ward in which she lived. This could have underestimated effects.

CONCLUSIONS

After four years, this government-led programme achieved improvements in life-saving interventions for mothers and newborns, demonstrating that even in settings where resources are constrained, the needle can be shifted in the right direction. We conclude that important and reproducible elements of this programme lie not for the most part in the individual components of the intervention, but in the way the programme was designed and implemented with its focus on government leadership and strong stakeholder partnership.

For peer review only

For peer review only - http://bmjopen.bmj.com/site/about/guidelines.xhtml

Acknowledgements

We are grateful to the women and families who participated and shared their experiences. We would like to thank all the health facility participants and birth attendants who took the time to contribute to our study. We would like to acknowledge Inuwa Jalingo from Data Research and Mapping Consult and recognise the contribution of Data Research and Mapping Consult field staff and supervisors who conducted the surveys. We would like to acknowledge the contribution of Vincent Ahonsi, Ester Agboa, and Tunde Segun from Evidence for Action, as well as Wale Adeleye from Champions for Change. Sincere thanks to all collaborators at the Gombe State Primary Health Care Development Agency, Gombe State Ministry of Health, Society for Family Health Nigeria, Pact Nigeria, Champions for Change, and Evidence for Action Mamaye. We would also like to acknowledge the contribution of our respected colleague and team member, Deepthi Wickremasinghe from the London School of Hygiene and Tropical Medicine, who sadly died on 2nd April 2020. Her wisdom, kindness and dedication to improving maternal and newborn health are greatly missed.

Contributions

BW conducted the analysis and drafted the manuscript. EB and EA provided statistical advice. BW, EB and JE verified the underlying data. NU, EB, AB, JE, KM, JS, NS and TM all provided important intellectual input. JA, ABB, MO, RS, AMG and AS coordinated study implementation and contributed to country data interpretation. UAU coordinated data collection activities and contributed to country data interpretation. TM and JS conceived the study. All authors have read, critically reviewed the manuscript and approved the final version of the manuscript.

Competing interests

All authors have completed the Unified Competing Interest form and declare no conflicts of interest.

Funding

The research was supported by IDEAS - Informed Decisions for Actions to improve maternal and newborn health (http://ideas.lshtm.ac.uk), which is funded through a grant from the Bill & Melinda Gates Foundation (Gates Global Health Grant Number: INV-007644) to the London School of Hygiene & Tropical Medicine.

Disclaimer

The funder of this study had no role in the study's design or conduct, data collection, analysis or interpretation of results, writing of the paper, or decision to submit for publication. The corresponding author had access to all study data and responsibility for the decision to submit the paper for publication.

Data sharing statement

De-identified participant datasets, protocols and questionnaires are uploaded to the LSHTM Data Repository. Data are available on reasonable request from the principal investigator of the IDEAS project and co-author for this manuscript, TM (ORCID id 0000-0002-4228-4334). Reuse permitted on request.

For peer review only - http://bmjopen.bmj.com/site/about/guidelines.xhtml

For peer review only

For peer review only - http://bmjopen.bmj.com/site/about/guidelines.xhtml

REFERENCES

1. Akseer N, Lawn JE, Keenan W, Konstantopoulos A, Cooper P, Ismail Z, et al. Ending preventable newborn deaths in a generation. International journal of gynaecology and obstetrics: the official organ of the International Federation of Gynaecology and Obstetrics. 2015;131 Suppl 1:S43-8.

2. GBD 2015 Maternal Mortality Collaborators. Global, regional, and national levels of maternal mortality, 1990-2015: a systematic analysis for the Global Burden of Disease Study 2015. Lancet (London, England). 2016;388(10053):1775-812.

3. PMNCH. A Global Review of the Key Interventions Related to Reproductive, Maternal, Newborn and Child Health (Rmnch). Geneva, Switzerland: The Partnership for Maternal, Newborn & Child Health. 2011.

4. Ronsmans C, Graham WJ. Maternal mortality: who, when, where, and why. Lancet (London, England). 2006;368(9542):1189-200.

5. WHO. Every newborn: an action plan to end preventable deaths. World Health Organization: Geneva. 2014.

6. WHO. Strategies towards ending preventable maternal mortality (EPMM). World Health Organization: Geneva. 2015.

7. Deorari A, Koganti RA. Closing the practice-to-outcome gap: lessons from the BetterBirth study. The Lancet Global health. 2019;7(8):e992-e3.

8. Gabrysch S, Nesbitt RC, Schoeps A, Hurt L, Soremekun S, Edmond K, et al. Does facility birth reduce maternal and perinatal mortality in Brong Ahafo, Ghana? A secondary analysis using data on 119 244 pregnancies from two cluster-randomised controlled trials. The Lancet Global health. 2019;7(8):e1074-e87.

9. Marx Delaney M, Miller KA, Bobanski L, Singh S, Kumar V, Karlage A, et al. Unpacking the null: a post-hoc analysis of a cluster-randomised controlled trial of the WHO Safe Childbirth Checklist in Uttar Pradesh, India (BetterBirth). The Lancet Global health. 2019;7(8):e1088-e96.

10. Alkema L, Chou D, Hogan D, Zhang S, Moller AB, Gemmill A, et al. Global, regional, and national levels and trends in maternal mortality between 1990 and 2015, with scenario-based projections to 2030: a systematic analysis by the UN Maternal Mortality Estimation Inter-Agency Group. Lancet (London, England). 2016;387(10017):462-74.

11. WHO. Trends in maternal mortality 2000 to 2017: estimates by WHO, UNICEF, UNFPA, World Bank Group and the United Nations Population Division. World Health Organization: Geneva. 2019.

12. Countdown to 2030 Collaboration. Countdown to 2030: tracking progress towards universal coverage for reproductive, maternal, newborn, and child health. Lancet (London, England). 2018;391(10129):1538-48.

13. UNICEF, WHO, World Bank, UN-DESA Population Division. Levels and trends in child mortality report 2019 Estimates developed by the UN Inter-agency Group for Child Mortality Estimation. United Nations Children's Fund: New York. 2019.

14. Amouzou A, Leslie HH, Ram M, Fox M, Jiwani SS, Requejo J, et al. Advances in the measurement of coverage for RMNCH and nutrition: from contact to effective coverage. BMJ global health. 2019;4(Suppl 4):e001297.

15. Carvajal-Aguirre L, Amouzou A, Mehra V, Ziqi M, Zaka N, Newby H. Gap between contact and content in maternal and newborn care: An analysis of data from 20 countries in sub-Saharan Africa. Journal of global health. 2017;7(2):020501.

16. Hodgins S, D'Agostino A. The quality-coverage gap in antenatal care: toward better measurement of effective coverage. Global health, science and practice. 2014;2(2):173-81.

17. Kruk ME, Gage AD, Arsenault C, Jordan K, Leslie HH, Roder-DeWan S, et al. High-quality health systems in the Sustainable Development Goals era: time for a revolution. The Lancet Global Health. 2018.

For peer review only

For peer review only - http://bmjopen.bmj.com/site/about/guidelines.xhtml

18. Kruk ME, Gage AD, Joseph NT, Danaei G, Garcia-Saiso S, Salomon JA. Mortality due to low-quality health systems in the universal health coverage era: a systematic analysis of amenable deaths in 137 countries. Lancet (London, England). 2018;392(10160):2203-12.
19. Ng M, Fullman N, Dieleman JL, Flaxman AD, Murray CJ, Lim SS. Effective coverage: a metric for monitoring Universal Health Coverage. PLoS Med. 2014;11(9):e1001730.
20. Yamey G. What are the barriers to scaling up health interventions in low and middle income countries? A qualitative study of academic leaders in implementation science. Globalization and health. 2012;8:11.
21. Wehrmeister FC, Fayé CM, da Silva ICM, Amouzou A, Ferreira LZ, Jiwani SS, et al. Wealth-related inequalities in the coverage of reproductive, maternal, newborn and child health interventions in 36 countries in the African Region. Bulletin of the World Health Organization. 2020;98(6):394-405.
22. Barros AJD, Wehrmeister FC, Ferreira LZ, Vidaletti LP, Hosseinpoor AR, Victora CG. Are the poorest poor being left behind? Estimating global inequalities in reproductive, maternal, newborn and child health. BMJ global health. 2020;5(1):e002229.
23. Gilson L, Lehmann U, Schneider H. Practicing governance towards equity in health systems: LMIC perspectives and experience. International journal for equity in health. 2017;16(1):171.
24. Spicer N, Hamza YA, Berhanu D, Gautham M, Schellenberg J, Tadesse F, et al. 'The development sector is a graveyard of pilot projects!' Six critical actions for externally funded implementers to foster scale-up of maternal and newborn health innovations in low and middle-income countries. Globalization and health. 2018;14(1):74.
25. National Population Commission and ICF. Nigeria Demographic and Health Survey 2018. Key Indicators Report. Abuja, Nigeria, and Rockville, Maryland, USA; 2019.
26. Izugbara CO, Wekesah FM, Adedini SA. Maternal Health in Nigeria. A Situation Update. Nairobi, Kenya: African Population and Health Research Centre; 2016.
27. National Bureau of Statistics (NBS) and United Nations Children's Fund (UNICEF). Multiple Indicator Cluster Survey 2016-17, Survey Findings Report. Abuja, Nigeria: National Bureau of Statistics and United Nations Children's Fund. 2017.
28. Bhattacharya AA, Umar N, Audu A, Felix H, Allen E, Schellenberg JRM, et al. Quality of routine facility data for monitoring priority maternal and newborn indicators in DHIS2: A case study from Gombe State, Nigeria. PloS one. 2019;14(1):e0211265.
29. Nigeria Health Management Information System, version 2013: Gombe State facility deliveries, by facility type, January-December 2018 [Internet]. Nigeria District Health Information System, version 2. 2019.
30. Gombe State Government. History of Gombe State, Nigeria. [cited 9 September 2019] Available from: http://gombestate.gov.ng/history-2/ 2018.
31. Gombe State Ministry of Health. GOMBE state government strategic health development plan 2010-2015. [cited on 19 August 2020] Available from: https://drive.google.com/file/d/0B1DAmtM1BcbMeVBRUjNEakViXzQ/view 2010.
32. WHO. Monitoring the Building Blocks of Health Systems: a handbook of indicators and their measurement strategies. World Health Organization: Geneva. 2010.
33. Habicht JP, Victora CG, Vaughan JP. Evaluation designs for adequacy, plausibility and probability of public health programme performance and impact. International journal of epidemiology. 1999;28(1):10-8.
34. The DHS Program. DHS Model Questionnaires. [cited 19 August 2020] Available from: https://www.dhsprogram.com/What-We-Do/Survey-Types/DHS-Questionnaires.cfm
35. Health Statistics and Information Systems, WHO. Service Availability and Readiness Assessment (SARA): an annual monitoring system for service delivery. Reference Manual, Version 2.2. World Health Organization: Geneva. 2015.
36. Filmer D, Pritchett LH. Estimating wealth effects without expenditure data--or tears: an application to educational enrollments in states of India. Demography. 2001;38(1):115-32.

For peer review only - http://bmjopen.bmj.com/site/about/guidelines.xhtml

37. Kruk ME, Leslie HH, Verguet S, Mbaruku GM, Adanu RMK, Langer A. Quality of basic maternal care functions in health facilities of five African countries: an analysis of national health system surveys. The Lancet Global health. 2016;4(11):e845-e55.
38. Marchant T, Beaumont E, Makowiecka K, Berhanu D, Tessema T, Gautham M, et al. Coverage and equity of maternal and newborn health care in rural Nigeria, Ethiopia and India. CMAJ : Canadian Medical Association journal = journal de l'Association medicale canadienne. 2019;191(43):E1179-e88.
39. Tomlin K, Berhanu D, Gautham M, Umar N, Schellenberg J, Wickremasinghe D, et al. Assessing capacity of health facilities to provide routine maternal and newborn care in low-income settings: what proportions are ready to provide good-quality care, and what proportions of women receive it? BMC Pregnancy Childbirth. 2020;20(1):289.
40. Marx Delaney M, Maji P, Kalita T, Kara N, Rana D, Kumar K, et al. Improving Adherence to Essential Birth Practices Using the WHO Safe Childbirth Checklist With Peer Coaching: Experience From 60 Public Health Facilities in Uttar Pradesh, India. Global health, science and practice. 2017;5(2):217-31.
41. Rowe AK, Rowe SY, Peters DH, Holloway KA, Chalker J, Ross-Degnan D. Effectiveness of strategies to improve health-care provider practices in low-income and middle-income countries: a systematic review. The Lancet Global health. 2018;6(11):e1163-e75.
42. The African Academy of Science, The Academy of Medical Science. From minding the gap to closing the gap: Science to transform maternal and newborn survival and stillbirths in sub-Saharan Africa in the Sustainable Development Goals era. Workshop Report 4–5 September 2018 Nairobi, Kenya. 2019.
43. Exley J, Hanson C, Umar N, Willey B, Shuaibu A, Marchant T. The provision of essential evidence-based interventions during facility-based childbirth: cross sectional observations of births in northeast Nigeria. BMJ Open. forthcoming.
44. Munabi-Babigumira S, Glenton C, Lewin S, Fretheim A, Nabudere H. Factors that influence the provision of intrapartum and postnatal care by skilled birth attendants in low- and middle-income countries: a qualitative evidence synthesis. The Cochrane database of systematic reviews. 2017;11(11):Cd011558.
45. Amare Y, Scheelbeek P, Schellenberg J, Berhanu D, Hill Z. Early postnatal home visits: a qualitative study of barriers and facilitators to achieving high coverage. BMC public health. 2018;18(1):1074.
46. Hill Z, Scheelbeek P, Hamza Y, Amare Y, Schellenberg J. Are We Using the Right Approach to Change Newborn Care Practices in the Community? Qualitative Evidence From Ethiopia and Northern Nigeria. Global health, science and practice. 2020.
47. Federal Government of Nigeria. Second National Strategic Health Development Plan 2018-2022. [cited 18 August 2020] Available from: http://www.health.gov.ng/doc/NSHDP%20II%20Final.pdf 2019.
48. Network for Improving Quality of Care for Maternal. Quality of Care in Nigeria. [cited 18 August 2020] Available from: http://www.qualityofcarenetwork.org/country-data/nigeria 2019.
49. Gilson L, Agyepong IA. Strengthening health system leadership for better governance: what does it take? Health Policy and Planning. 2018;33(suppl_2):ii1-ii4.
50. Bulthuis SE, Kok MC, Raven J, Dieleman MA. Factors influencing the scale-up of public health interventions in low- and middle-income countries: a qualitative systematic literature review. Health Policy Plan. 2020;35(2):219-34.

For peer review only - http://bmjopen.bmj.com/site/about/guidelines.xhtml

BMJ Open

Improving maternal and newborn health services in northeast Nigeria through a government-led partnership of stakeholders: a quasi-experimental study

Journal:	*BMJ Open*
Manuscript ID	bmjopen-2021-048877.R1
Article Type:	Original research
Date Submitted by the Author:	25-Aug-2021
Complete List of Authors:	Willey, Barbara; London School of Hygiene & Tropical Medicine, Department of Infectious Disease Epidemiology<br>UMAR, NASIR; London School of Hygiene & Tropical Medicine, Department of Disease Control<br>Beaumont, Emma; London School of Hygiene & Tropical Medicine, Department of Infectious Disease Epidemiology<br>Allen, Elizabeth; London School of Hygiene & Tropical Medicine, Department of Medical Statistics<br>Anyanti, Jennifer; Society for Family Health Nigeria<br>Bello, Abubakar Bala ; PACT<br>Bhattacharya, Antoinette Alas; London School of Hygiene & Tropical Medicine, Department of Disease Control<br>Exley, Josephine; London School of Hygiene & Tropical Medicine, Department of Disease Control<br>Makowiecka, Krystyna; London School of Hygiene & Tropical Medicine, Department of Infectious Disease Epidemiology<br>Okolo, Magdalyn; Society for Family Health Nigeria<br>Sani, Rabi; PACT<br>Schellenberg, Joanna; London School of Hygiene and Tropical Medicine, Department of Disease Control<br>Spicer, Neil; London School of Hygiene & Tropical Medicine, Department of Global Health and Development<br>Usman, Umar Adamu; Data Research and Mapping Consult LTD<br>Gana, Ahmed; Gombe State Primary Health Care Development Agency<br>Shuaibu, Abdulrahman ; State Primary Health Care Development Agency Gombe State, Nigeria, GSPHCDA<br>Marchant, Tanya; London School of Hygiene & Tropical Medicine, Department of Disease Control
**Primary Subject Heading**:	Health policy
Secondary Subject Heading:	Public health, Health policy, Global health
Keywords:	Organisation of health services < HEALTH SERVICES ADMINISTRATION & MANAGEMENT, Public health < INFECTIOUS DISEASES, NEONATOLOGY, Maternal medicine < OBSTETRICS

SCHOLARONE™
Manuscripts

I, the Submitting Author has the right to grant and does grant on behalf of all authors of the Work (as defined in the below author licence), an exclusive licence and/or a non-exclusive licence for contributions from authors who are: i) UK Crown employees; ii) where BMJ has agreed a CC-BY licence shall apply, and/or iii) in accordance with the terms applicable for US Federal Government officers or employees acting as part of their official duties; on a worldwide, perpetual, irrevocable, royalty-free basis to BMJ Publishing Group Ltd ("BMJ") its licensees and where the relevant Journal is co-owned by BMJ to the co-owners of the Journal, to publish the Work in this journal and any other BMJ products and to exploit all rights, as set out in our licence.

The Submitting Author accepts and understands that any supply made under these terms is made by BMJ to the Submitting Author unless you are acting as an employee on behalf of your employer or a postgraduate student of an affiliated institution which is paying any applicable article publishing charge ("APC") for Open Access articles. Where the Submitting Author wishes to make the Work available on an Open Access basis (and intends to pay the relevant APC), the terms of reuse of such Open Access shall be governed by a Creative Commons licence – details of these licences and which Creative Commons licence will apply to this Work are set out in our licence referred to above.

Other than as permitted in any relevant BMJ Author's Self Archiving Policies, I confirm this Work has not been accepted for publication elsewhere, is not being considered for publication elsewhere and does not duplicate material already published. I confirm all authors consent to publication of this Work and authorise the granting of this licence.

For peer review only

Improving maternal and newborn health services in northeast Nigeria through a government-led partnership of stakeholders: a quasi-experimental study

Barbara Willey[1], Nasir Umar[1], Emma Beaumont[1], Elizabeth Allen[1], Jennifer Anyanti[4], Abubakar Bala Bello[3], Antoinette Bhattacharya[1], Josephine Exley[1], Krystyna Makowiecka[1], Magdalyn Okolo[4], Rabi Sani[3], Joanna Schellenberg[1], Neil Spicer[1], Umar Adamu Usman[2], Ahmed Mohammed Gana[5], Abdulrahman Shuaibu[6], Tanya Marchant[1]

Affiliations

[1] London School of Hygiene & Tropical Medicine, London, UK

[2] Data Research and Mapping Consult Limited, Abuja, Nigeria

[3] Pact, Abuja, Nigeria

[4] Society for Family Health, Abuja, Nigeria

[5] Office of the Honourable Commissioner for Health (and former Executive Secretary GSPCDA), Gombe State Ministry, Gombe, Nigeria

[6] Office of the Executive Secretary, Gombe State Primary Health Care Development Agency, Gombe, Nigeria

Corresponding author email:

Barbara.Willey@lshtm.ac.uk

For peer review only

Abstract

Objectives

This study aimed to quantify change in the coverage, quality and equity of essential maternal and newborn health care interventions in Gombe state, northeast Nigeria, following a four year, government-led, maternal and newborn health intervention.

Design

Quasi-experimental plausibility study. Repeat cross-sectional household and linked health facility surveys were implemented in intervention and comparison areas.

Setting

Gombe State, northeast Nigeria.

Participants

Each household survey included a sample of 1,000 women aged 13-49 years with a live birth in the previous twelve months. Health facility surveys comprised a readiness assessment and birth attendant interview.

Interventions

Between 2016-2019 a complex package of evidence-based interventions was implemented to increase access, use and quality of maternal and newborn health care, spanning the six WHO health system building blocks.

Outcome measures

Eighteen indicators of maternal and newborn health care.

Results

Between 2016 and 2019, the coverage of all indicators improved in intervention areas, with the exception of post-natal and post-partum contacts, which remained below fifteen percent. Greater improvements were observed in intervention than comparison areas for eight indicators, including coverage of at least one antenatal visit (71% (95% CI 62-68) to 88% (95% CI 82-93)), at least four antenatal visits (46% (95% CI 39-53) to 69% (95% CI 60-75)), facility birth (48% (95% CI 37-59) to 64% (95% CI 54-73)), administration of uterotonics (44% (95% CI 34-54) to 59% (95% CI 50-67)), delayed newborn bathing (44% (95% CI 36-52) to 62% (95% CI 52-71)), and clean cord care (42% (95% CI 34-49) to 73% (95% CI 66-79)). Wide-spread inequities persisted however; only at least one antenatal visit saw pro-poor improvement.

Conclusions

This intervention achieved improvements in life-saving behaviours for mothers and newborns, demonstrating that multi-partner action, coordinated through government leadership, can shift the needle in the right direction, even in resource constrained settings.

For peer review only - http://bmjopen.bmj.com/site/about/guidelines.xhtml

Strengths and limitations of the study

- This was a large, multi-year study.
- Tools measured access, use, quality and equity of maternal and newborn health care in a setting with a high mortality burden.
- Standardised tools were applied each year to collect both population level and facility level evidence.
- The selection of intervention and comparison areas was pragmatic, following government implementation plans.
- In this non-randomised design, baseline characteristics between areas were not balanced, and residual confounding by area cannot be discounted.

For peer review only

For peer review only - http://bmjopen.bmj.com/site/about/guidelines.xhtml

INTRODUCTION

Interventions to reduce maternal and newborn morbidity and mortality are available (1-6), however, implementation gaps create a mismatch between the efficacy and effectiveness of interventions at a population level (7-9). Despite substantial progress in some regions during the era of the Millennium Development Goals (10), maternal and neonatal mortality remain unacceptably high, especially in low- and middle-income countries (11-13). The era of Universal Health Coverage highlights the need for a focus on quality of care and reducing inequalities alongside increasing access to care (14-19).

Challenges remain in how to equitably scale and package efficacious interventions and integrate them within existing health systems (20). Examining maternal, newborn, and child health coverage data from 36 sub-Saharan African countries, Wehrmeister and colleagues concluded that countries had narrowed equity gaps since 1995, although wealth-related inequalities remain highest in West Africa. (21). In their study of 83 low- and middle-income countries, Barros and colleagues emphasised the need for implementation of pro-poor strategies if the most vulnerable are to benefit from available health care (22). Part of the solution to these challenges may be robust governance and government-led initiatives that focus on universal and equitable access (23, 24).

Here we report the effect of the Gombe Partnership for Maternal and Newborn Health, a government-led intervention implemented between 2016 and 2019 in north-eastern Nigeria and designed to coordinate multiple actors toward the goal of equitably improving high quality maternal and newborn health services. An intended outcome of the partnership was to inform scale up and share learning across neighbouring states and regions. Implementation used an adaptive management approach and partners met regularly to examine monitoring data and amend implementation as necessary. In this paper, we examine the effect of this intervention on the coverage and quality of essential maternal and newborn health care interventions after four years of implementation, and assess whether any improvements were equitable across socio-economic groups.

For peer review only - http://bmjopen.bmj.com/site/about/guidelines.xhtml

METHODS

Setting and intervention implementation

This study was conducted in Gombe State, a predominately rural (80%) and sparsely-populated state in north-eastern Nigeria (25), where the burden of maternal and neonatal mortality is higher than the national average and stood at 1,549 maternal deaths per 100,000 live births in 2015 and 33 neonatal deaths per 1,000 live births in 2017 (26, 27). In 2016, when this study started, 29% of women gave birth in a health facility (25), principally at primary health centres (PHC) (28, 29). Primary health care services are predominately delivered by the Gombe State Primary Health Care Development Agency, with little private sector provision, unlike some other regions of Nigeria (30). In 2016, 460 PHCs in the state provided antenatal care (ANC), birth and intrapartum services (28), mainly delivered by community health extension workers (CHEWs), junior CHEWs and community health officers, but very few nurses, midwives or doctors (31).

From 2016-2019, the government led a maternal and newborn health partnership to improve access, use and quality of maternal and newborn health services. Within this partnership, non-governmental organisations (NGOs) implemented a package of evidence-based interventions that spanned the six WHO health system building blocks (32) (figure 1). Components aimed to enhance uptake and provision of life-saving interventions at three interacting levels (individuals and families; community organisations; and the health system). At the individual and family level, interventions aimed to improve knowledge, attitudes and practices to increase enhanced home-based practices and increase demand for routine professional care; for example, a community-based Village Health Worker home visit scheme was initiated to improve knowledge about and linkages between families and health services (33). At the community organisation level, interventions aimed to improve trust and accountability between the family and health system levels; for example through supporting community-based Mothers Groups to interact with their local primary health services (34). Interventions at the health system level aimed to improve the supply of safe, effective and high quality care; for example, working with government to strengthen the supply chain for essential drugs in PHCs. Underpinning these three levels of engagement were interventions designed to raise public awareness about maternal and newborn health across the state through mass media and advocacy events (35).

This package of interventions was deliberately coordinated by government as a pathway towards improved maternal and newborn outcomes (36). The government, NGOs, partners and the funder met six-monthly to review monitoring data, trouble-shoot implementation challenges, course-correct, and reinvigorate communal purpose towards a shared goal. To facilitate learning, the package of interventions was initially implemented in an intervention area, with a view to scaling-up to the entire state. The intervention area was defined as 57 sub-district level wards (half of the state's 114 wards), purposively identified by government. Community-based demand generation activities were implemented in these wards, and one centrally located PHC within each ward was chosen to implement the activities designed to improve health service quality: the rationale being that it was preferable to have one well-functioning PHC per ward, rather than a larger number of less well-functioning facilities. Residents of the state's remaining 57 wards (comparison area) continued to receive their usual care, with the exception of mass media components which were state-wide (figure 1).

For peer review only - http://bmjopen.bmj.com/site/about/guidelines.xhtml

Study design

We used a quasi-experimental plausibility study design (37) to explore the association between the intervention and indicators of use and quality of maternal and newborn health services, comparing changes observed over time in intervention areas to those in comparison areas. Repeat cross-sectional household and linked health facility surveys were undertaken. Survey methods were replicated each time. Data collection tools were informed by existing large scale survey tools such as the Demographic and Health Surveys (38) and Service Availability and Readiness Assessment Surveys (39).

We conducted four annual household surveys during July/August 2016-2019. The household survey consisted of a modular household questionnaire: (1) A household module which asked about characteristics of the household and ownership of commodities as proxy markers of household socioeconomic status, and during which a household roster of all usually resident people was generated; (2) A women's module which asked all resident women about the health care available to them, their recent contact with health services and their recent birth history; and (3) A mother's module which asked all women who reported a birth in the last 12 months about their contact with health services across the continuum of care from pregnancy to postnatal care. In a household with a resident recently delivered woman the questionnaire took approximately 90 minutes to complete.

A random sample of 80 clusters was selected: 40 each from intervention and comparison wards. Clusters were segmented enumeration areas as defined by the National Population Commission. Within each cluster, all households were visited. During each survey, we aimed to survey a total of 6,000 households across the 80 clusters. This was expected to result in interviews with 1,000 women with a live birth in the previous 12 months (i.e. 500 each in intervention and comparison). Sample size calculations assumed a design effect of 2.5, 95% probability and 80% power. For each survey, where comparison prevalence of indicators ranged between 20 and 60 percent, this sample size was sufficient to detect changes of 15 percentage points in intervention areas.

During the same period, we conducted seven facility and birth attendant surveys at six-monthly intervals. Four surveys were done concurrently with the household surveys (figure 2). At each time point, a facility readiness assessment was carried out plus an interview was conducted with the available birth attendant who had attended the most recent delivery recorded in the maternity register. The facility survey took approximately 120 minutes to complete all sections. All 57 intervention PHCs were surveyed plus one PHC selected at random from each of the 40 comparison clusters sampled during household surveys. For each survey, where prevalence of indicators in comparison facilities ranged between 5 and 70 percent, this sample of 97 facilities was sufficient to detect changes of 20 percentage points in intervention areas.

All recruited interviewers were from Gombe State and attended a one-week training course at each survey. Eight survey teams were recruited in total including a supervisor, four household interviewers, one facility and birth attendant interviewer, one mapper who listed households and segmented enumeration areas as necessary, and one data support member. Questionnaires were translated and back-translated between English and Hausa languages to ensure consistency and were pre-tested. As part of the week long training, the full study protocol was pilot tested in two clusters to identify and correct any operational or language problems. All data were collected using hand-held digital devices and synchronized with a supervisor laptop each day. Automated summary reports were produced to identify and address any internal inconsistencies.

For peer review only - http://bmjopen.bmj.com/site/about/guidelines.xhtml

Statistical analysis of effectiveness and equity

Eighteen indicators measuring the coverage of life-saving commodities and behaviours were selected *a priori* for their known association with maternal and newborn health outcomes (box 1). Changes over the four-year study period were compared between intervention and comparison areas. Analyses were at the individual level for household survey indicators and at the health facility level for facility and birth attendant indicators. Data were analysed in Stata 15 (StataCorp, 2017, College Station, TX).

Household and health facility characteristics were summarised using appropriate summary statistics. We used survey commands (*svy*) to account for clustering. We identified variables which differed significantly between intervention and comparison areas in 2016 using a design-based F test and included their cluster-level means in regression models. Percentage point differences and 95% CIs in indicator coverage between 2016 and 2019 were calculated.

Effectiveness of the programme on pre-specified indicators was estimated through logistic regression models. A likelihood ratio test comparing models with time as a continuous variable to one where each time point was included separately, determined whether time was included as a continuous or categorical variable. Models included fixed effects for area (intervention vs comparison), time (at all time points) and the interaction between area and time, to describe any additional effect in 2019 in the intervention areas compared to 2016 in the comparison areas. Cluster level means of variables which differed between intervention and comparison areas in 2016, identified using a design-based F-test, were included in all models. We included a random effect for cluster to account for clustering in logistic regression models of household survey indicators.

To examine equity, principal components analysis was used to generate an index of household wealth, based on asset ownership (36). Using this, households were categorised into quintiles from poorest to least poor. Where there was evidence of greater improvement in the intervention areas compared to the comparison areas, we examined the difference of change over time by household socioeconomic quintile in the intervention areas. We tested for interaction between time and socioeconomic status quintile.

Ethics

This research was conducted with approval from: The Nigerian National Health Research Ethics Committee (NHREC/01/01/2007); the State Ministry of Health Gombe State (ADM/S/658/Vol. II/66); and the London School of Hygiene & Tropical Medicine (12181). The free and informed written consent of all participants was obtained.

Patient and public involvement

Patients and the public were not involved in the design, conduct, reporting or dissemination plans of our research.

For peer review only - http://bmjopen.bmj.com/site/about/guidelines.xhtml

Box 1: Indicator definitions

Indicator name	Numerator	Denominator
	Antenatal care period	
Women made at least one ANC visit	Number of women aged 15-49 years with a birth during 12 months before survey who made at least one ANC visit	Women aged 15-49 years with a birth during 12 months before survey
Women made at least four ANC visits	Number of women aged 15-49 years with a birth during 12 months before survey who made at least four ANC visits	Women aged 15-49 years with a birth during 12 months before survey
Women received basic ANC quality	Number of women aged 15-49 years with a birth during 12 months before survey attending ANC who received all three of: urine test, blood test, and blood pressure measurement	Women aged 15-49 years with a birth during 12 months before survey
Women received syphilis test results	Number of women aged 15-49 years with a birth during 12 months before survey who received a test result for syphilis	Women aged 15-49 years with a birth during 12 months before survey
Facilities had syphilis treatment available	Number of primary health facilities which had benzathine penicillin for syphilis treatment in stock	Primary health facilities surveyed
	Intrapartum care period	
Facility delivery	Number of women aged 15-49 years with a birth during 12 months before survey who gave birth in a health facility	Women aged 15-49 years with a birth during 12 months before survey
Uterotonics	Number of women aged 15-49 years with a birth during 12 months before survey who received prophylactic uterotonics, includes facility and home births	Women aged 15-49 years with a birth during 12 months before survey
Facilities had pre-eclampsia treatment available	Number of primary health facilities which had magnesium sulphate available for treatment of pre-eclampsia	Primary health facilities surveyed
Birth attendant was able to correctly manage pre-eclampsia	Number of birth attendants who were able to correctly manage pre-eclampsia, as assessed by clinical vignette	Birth attendants interviewed
	Immediate newborn care period	
Facilities had newborn resuscitation equipment available	Number of primary health facilities which had a bag and mask for newborn resuscitation (size 0 and size 1)	Primary health facilities surveyed
Birth attendant was able to correctly manage newborn resuscitation	Number of birth attendants who were able to correctly manage neonatal resuscitation, as assessed by simulation	Birth attendants interviewed
Newborn bathing was delayed until at least 24 hours after birth	Number of newborns born to women aged 15-49 years with a birth during 12 months before survey who had delayed bathing for the first 24 hours of life	Women aged 15-49 years with a birth during 12 months before survey
Newborn received clean cord care	Number of newborns women aged 15-49 years with a birth during 12 months before survey who received clean cord care, defined as cutting cord with clean blade or scissors, administering chlorhexidine to the newborn	Women aged 15-49 years with a birth during 12 months before survey

For peer review only

For peer review only - http://bmjopen.bmj.com/site/about/guidelines.xhtml

Indicator name	Numerator	Denominator
	cord, and not administering anything harmful to the newborn cord	
Newborn was breastfed within 1 hour of birth	Number of newborns born to women aged 15-49 years with a birth during 12 months before survey for which breastfeeding was initiated within 1 hour of birth	Women aged 15-49 years with a birth during 12 months before survey
	Post-partum and post-natal care period	
Women received a post-partum check within 2 days of delivery	Number of women aged 15-49 years with a birth during 12 months before survey who had a post-partum check-up within 2 days of delivery	Women aged 15-49 years with a birth during 12 months before survey
Newborns received a post-natal check within 2 days of birth	Number of newborns born to women aged 15-49 years with a birth during 12 months before survey who had a post-natal check-up within 2 days of birth	Women aged 15-49 years with a birth during 12 months before survey
Newborns with suspected sepsis treated with antibiotics	Number of newborns aged 0-60 days with suspected sepsis who received antibiotics	Newborns aged 0-60 days with suspected sepsis
Facilities had newborn sepsis treatment available	Number of primary health facilities with amoxicillin and gentamicin available	Primary health facilities surveyed

Note: data were collected for women aged 13–49 years. However, no women <15 years of age had a live birth in the 12 months before the survey. Antenatal care (ANC).

For peer review only

RESULTS

Sample description

Table 1 shows the number of households, resident women, and facilities surveyed. High response rates were seen throughout. Table 2 presents baseline characteristics for the 1,011 women interviewed with a recent live birth. Women had a mean age of 26 years (SD 0.4) and parity of four births (SD 0.2); over one quarter reported more than six births. Marriage was near universal (>94%) and 82% of women were Muslim. Mean year of schooling was 4.3 (SD 0.6) in intervention and 2.6 years (SD 0.5) in comparison (p=0.06). Although the majority reported no formal education, this was substantially higher in comparison areas (72% vs. 55%, p=0.05).

Table 1: Interviews conducted, by survey and year

	2016	2017	2018	2019
Number of clusters	80	80	80	80
Households				
Target number of households to be surveyed	6000	6000	6000	6000
Number of households surveyed	5747	5762	5925	5616
Response rate	96%	96%	99%	94%
Resident women				
Number of resident women aged 15-49 years	8556	8395	10177	10080
Number of women interviewed	8453	8270	10150	9959
Response rate	99%	98%	99%	99%
Number of resident women with live birth in 12 months preceding survey	1011	932	991	871
Primary health facilities				
Target number of primary health facilities to be surveyed	97	97	97	97
Number of primary health facilities surveyed	94	94	97	97
Response rate	97%	97%	100%	100%
Number of birth attendants interviewed (one per facility)	92	91	93	95
Response rate	95%	94%	96%	98%

Note: The survey sampled resident women aged 13-49 years, however, no births in the last twelve months were recorded among women aged 13-14 years and as such the sample of women with a recent birth is aged 15-49 years.

For peer review only

For peer review only - http://bmjopen.bmj.com/site/about/guidelines.xhtml

Table 2: Baseline characteristics: women with recent birth (2016 survey)

	Intervention		Comparison		p-value**
	N=519	% (95% CI)	N=492	% (95% CI)	
Woman's age category					
15-19 years	54	10 (8-14)	60	12 (9-16)	
20-24 years	163	32 (27-37)	128	26 (23-30)	
25-29 years	146	28 (25-32)	128	26 (22-31)	
30-34 years	87	17 (14-20)	94	19 (16-23)	0.35
35-39 years	48	9 (7-12)	56	11 (9-14)	
40-44 years	15	3 (2-5)	21	4 (3-7)	
45-49 years	3	0.6 (0.2-2)	4	0.8 (0.3-2)	
Parity					
1 birth	111	21 (18-26)	79	16 (13-20)	
2 births	76	15 (11-19)	83	17 (14-20)	0.07
3-5 births	205	39 (35-44)	178	37 (32-41)	
≥ 6 births	127	25 (20-30)	152	31 (27-35)	
Marital status					
Currently married	488	94 (89-97)	472	96 (93-98)	0.35
Not married	31	6 (3-10)	20	4 (2-7)	
Religion					
Christianity	92	18 (10-31)	71	14 (8-25)	0.62
Islam	426	82 (69-90)	421	86 (75-92)	
Education level completed					
None	283	55 (42-67)	353	72 (60-81)	
1-7 years (primary)	87	17 (11-24)	56	11 (7-19)	0.05
≥ 8 years (secondary)	149	29 (20-39)	83	17 (11-26)	
*Household socioeconomic status, by quintile**					
Quintile 1 (poorest)	102	20 (14-27)	113	23 (15-33)	
Quintile 2	96	19 (14-24)	111	23 (17-29)	
Quintile 3	114	22 (17-27)	96	20 (15-24)	0.44
Quintile 4	94	18 (14-24)	100	20 (15-27)	
Quintile 5 (richest)	113	22 (14-33)	72	15 (9-23)	

* Socioeconomic asset index: wall material not thatch or mud, floor material not earth or sand, clean source of drinking water, roof material iron, tiles or cement, flush toilet or latrine, electricity supply to home, ownership of fridge, TV, radio, bike, kerosene lamp, wrist watch, motorcycle, generator, fan. ** p-value from design-based F test

For peer review only

For peer review only - http://bmjopen.bmj.com/site/about/guidelines.xhtml

Table 3 shows that in 2016, almost all sampled facilities provided ANC and delivery services, and at least 70% of facilities provided postnatal care. Only a small minority of facilities had a trained midwife available 24 hours a day. In total, a third of intervention PHCs, and a quarter of comparison PHCs provided all basic emergency obstetric and newborn care (BEmONC) signal functions (excluding assisted vaginal delivery) in the previous three months (p=0.58). Facilities had low volumes of deliveries (<500 births per year) (40), with each intervention PHC managing a mean of 288 births per year versus 192 in comparison PHCs. In intervention PHCs 55 birth attendant were interviewed, and 37 in comparison PHCs. The cadre of the interviewed birth attendant was similar between intervention and comparison PHCs. Very few were nurses or midwives (4% in intervention, none in comparison), and approximately half in both areas were community health extension workers, while the remaining 50% in each area were non-clinical health workers. These birth attendants were similar in age, a mean of 36 years (SD 1.0) in intervention and of 39 years (SD 1.3) in comparison PHCs, and both had an average of 13 years' education (SD 0.6). On average, duration in post was five years (SD 0.6) in intervention and four years (SD 0.7) in comparison PHCs, suggesting relatively stable staff turnover.

Table 3: Baseline characteristics: health facilities (2016 survey)

	Intervention		Comparison		p-value *
	N=55	(%, 95% CI)	N=39	(%, 95% CI)	
Infrastructure					
Clean source of running water	34	62 (48-74)	17	44 (29-60)	0.09
Electricity connection	38	69 (56-80)	24	62 (45-76)	0.45
Opening hours					
7 days a week	44	80 (67-89)	25	68 (51-81)	0.18
Services offered					
ANC services available	55	100	37	95 (81-99)	0.09
Delivery services available	55	100	34	87 (72-95)	0.01
Post-natal care services available	42	76 (63-86)	27	69 (53-82)	0.45
24hour delivery services available 7 days a week	44	83 (70-91)	27	82 (65-92)	0.08
Trained midwife available 24 hours a day 7 days a week	14	15 (9-24)	12	13 (7-21)	0.57
Provided all BEmONC signal functions (excluding assisted vaginal delivery) in previous 3 months	17	31 (20-45)	10	26 (14-42)	0.58

Antenatal care (ANC), Basic emergency obstetric and newborn care (BEmONC). * p-value from design-based F test

For peer review only - http://bmjopen.bmj.com/site/about/guidelines.xhtml

Change over time in intervention and comparison areas

Table 4 shows prevalence of 18 maternal and newborn health indicators between 2016 and 2019 in intervention and comparison areas for women with a recent live birth, PHCs, and surveyed birth attendants. With the exception of post-partum and post-natal health checks, which did not improve, 16 indicators improved over time in the intervention area (table 4).

Table 4 shows that seven indicators improved in the intervention areas only, while nine also improved in comparison areas. For eight indicators, greater changes over time were observed in intervention areas above any increases seen in comparison areas. The proportion of women attending ANC visits and giving birth in a facility increased to 88% for ANC1, 69% for ANC4 and 64% for facility birth. These represented improvements up to nine percentage points above increases seen in comparison areas (p=0.006 and p=0.003, respectively for ANC1 and ANC4, and p<0.001 for facility birth). For the intrapartum period, we saw an increase in the administration of prophylactic uterotonics among all women reaching 59%, a four percentage point improvement above changes in the comparison areas (p=0.008). Availability of treatment for pre-eclampsia increased by 32 percentage points above changes in the comparison areas (p=0.021). Adoption of life-saving behaviours for immediate newborn care also increased, with improvements up to 10 percentage points higher than changes seen in comparison areas (p=0.034 for delayed bathing of the newborn, and p<0.001 for clean cord care, respectively). Availability of newborn bag and mask for resuscitation increased by 36 percentage points above changes in the comparison areas (p=0.001). Similar improvements were observed in intervention and comparison areas for three indicators: women receiving syphilis test results (p=0.758); birth attendants' capability to manage pre-eclampsia appropriately (p=0.971), and immediate breastfeeding of the newborn (p=0.524) (table 4).

For peer review only - http://bmjopen.bmj.com/site/about/guidelines.xhtml

Table 4: Indicators over time by intervention and comparison area

Indicator	Area	2016			2017			2018			2019			Percentage point difference 2019-2016 (95% CI) p-value*	Linear trend p-value**	p-value interaction time and area ***
		N	n	% (CI)	N	n	% (CI)	N	n	% (CI)	N	n	% (CI)			
**Antenatal care period**																
Women made at least one ANC visit	Intervention	519	366	71 (62-78)	494	350	71 (64-77)	511	435	85 (79-90)	458	405	88 (82-93)	17 (11-25) <0.001	<0.001	0.006
	Comparison	492	279	57 (47-66)	438	239	55 (46, 63)	480	292	61 (51-70)	413	298	72 (61-81)	15 (6-25) 0.002	0.014	
Women made at least four ANC visits	Intervention	519	239	46 (39-53)	494	230	46 (39-53)	511	326	64 (56-71)	511	315	69 (60-76)	23 (14-32) <0.001	0.002	0.003
	Comparison	493	145	29 (23-37)	438	131	30 (23-37)	480	164	34 (25-45)	480	179	43 (34-53)	14 (6-22) <0.001	0.458	
Women received basic ANC quality	Intervention	519	303	58 (50-66)	494	261	53 (44-62)	511	312	61 (52-69)	458	305	66 (58-74)	8 (1-18) 0.008	0.002	0.599
	Comparison	492	216	44 (35-53)	438	160	37 (29-45)	480	183	38 (30-47)	413	220	53 (42, 64)	9 (-0.2-19) 0.056	NA	
Women received syphilis test results	Intervention	519	61	12 (9-16)	494	86	17 (12-24)	511	166	32 (26-40)	458	149	33 (27-38)	21 (14-27) <0.001	<0.001	0.758
	Comparison	492	39	8 (6-11)	438	55	13 (8-20)	480	96	20 (14-27)	413	88	21 (15-30)	13 (5-21) 0.002	0.008	
Facilities PHC had syphilis treatment available	Intervention	55	5	9 (4-20)	55	29	53 (39-66)	57	37	65 (52-76)	57	21	37 (25-50)	28 (12-44) <0.001	<0.001	0.061
	Comparison	39	3	8 (2-22)	39	7	18 (9-33)	40	6	15 (7-30)	41	5	12 (5-26)	4 (-8-17) 0.467	NA	
**Intrapartum care period**																
Women gave birth in a facility	Intervention	519	248	48 (37-59)	494	217	44 (33-55)	511	313	61 (53-69)	458	294	64 (54-73)	16 (7-26) 0.002	<0.001	<0.001
	Comparison	492	128	26 (18-35)	436	154	35 (27-45)	480	147	31 (23-39)	412	145	35 (26-46)	9 (0.5-18) 0.039	0.078	
Women received uterotonic	Intervention	509	223	44 (34-54)	473	186	39 (30-49)	505	264	52 (45-60)	435	256	59 (50-67)	15 (6-24) 0.001	0.002	0.008
	Comparison	487	133	27 (20-37)	416	127	31 (23-40)	475	132	28 (21-35)	372	141	38 (29-48)	11 (3-19) 0.010	0.527	
PHC had pre-eclampsia treatment available	Intervention	55	11	20 (11-33)	55	35	64 (51-75)	57	56	98 (88-100)	57	53	93 (82-97)	73 (60-86) <0.001	<0.001	0.021
	Comparison	39	5	13 (5-28)	39	7	18 (9-33)	40	23	58 (42-72)	41	22	54 (38-68)	41 (24-58) <0.001	0.062	
Birth attendant was able to correctly	Intervention	55	16	29 (18-43)	55	23	42 (29-55)	57	46	81 (68-89)	57	50	88 (76-94)	59 (42-75) <0.001	0.023	0.971

For peer review only - http://bmjopen.bmj.com/site/about/guidelines.xhtml

manage pre-eclampsia	Comparison	37	10	27 (15-44)	36	11	31 (18-48)	36	19	53 (36-69)	38	27	71 (55-83)	44 (24-64) <0.001	0.005	
**Immediate newborn care period**																
Facilities had newborn resuscitation equipment available	Intervention	55	35	64 (50-75)	55	45	82 (69-90)	57	51	89 (78-95)	57	54	95 (85-98)	31 (17-46) <0.001	<0.001	0.001
	Comparison	39	17	44 (29-60)	39	12	31 (18-47)	40	8	20 (10-35)	41	16	39 (25-55)	-5 (-18-9) 0.509	NA	
Birth attendant was able to correctly manage newborn resuscitation	Intervention	55	6	11 (5-23)	55	12	22 (13-35)	57	27	47 (35-60)	57	27	47 (35-60)	36 (19-54) <0.001	0.038	0.775
	Comparison	37	5	14 (6-29)	36	5	14 (6-30)	36	8	22 (11-39)	38	9	24 (13-40)	10 (-8-29) 0.276	NA	
Newborn bathing was delayed until at least 24 hours after birth	Intervention	519	226	44 (36-52)	492	224	46 (37-54)	511	305	60 (50-69)	458	285	62 (52-71)	18 (8-30) 0.002	0.012	0.034
	Comparison	492	161	33 (26-40)	435	136	31 (24-39)	480	188	39 (30-49)	412	169	41 (32-51)	8 (-1-18) 0.090	NA	
Newborn received clean cord care	Intervention	519	216	42 (34-49)	494	212	43 (36-50)	511	331	65 (57-72)	458	335	73 (66-79)	31 (21-42) <0.001	<0.001	<0.001
	Comparison	492	140	28 (23-35)	438	134	31 (24-38)	480	159	33 (26-41)	413	208	50 (41-59)	22 (13-31) <0.001	0.028	
Newborn was breastfed within 1 hour of birth	Intervention	511	270	53 (46-60)	489	283	58 (49-66)	509	531	69 (62-75)	454	304	67 (59-74)	14 (3-25) 0.016	0.028	0.524
	Comparison	483	215	45 (39-50)	430	207	48 (40-56)	474	262	55 (49-62)	409	249	61 (51-70)	16 (7-26) 0.002	0.586	
**Post-partum and post-natal care period**																
Women received a post-partum check within 2 days of delivery	Intervention	519	56	11 (7-17)	492	39	8 (5-12)	507	59	12 (8-17)	457	60	13 (9-19)	2 (-4-9) 0.451	NA	0.018
	Comparison	492	24	5 (3-9)	434	23	5 (3-8)	480	11	2 (1-5)	413	34	8 (5-13)	3 (1-8) 0.002	0.002	
Newborns received a post-natal check within 2 days of birth	Intervention	519	28	5 (3-9)	491	27	5 (3-10)	510	32	6 (4-10)	458	31	7 (4-11)	2 (-2- 5) 0.391	NA	0.059
	Comparison	492	18	4 (2-7)	435	10	2 (1-4)	479	5	1 (0.3-3)	412	17	4 (2-10)	0 (-4-4) 0.825	NA	
Newborns with suspected sepsis treated with antibiotics	Intervention	43	15	35 (22-50)	33	13	39 (23-58)	25	9	36 (15-64)	15	11	73 (44-91)	38 (12-65) 0.006	0.198	0.273
	Comparison	29	13	45 (28-62)	23	9	39 (24-57)	20	4	20 (9-38)	21	10	48 (26-70)	3 (-27, 33) 0.852	NA	
Facilities had newborn sepsis treatment available	Intervention	55	20	36 (25-50)	55	38	69 (56-80)	57	36	63 (50-75)	57	38	67 (53-78)	31 (10-50) 0.004	<0.001	0.054
	Comparison	39	12	31 (18-47)	39	15	38 (24-55)	40	9	23 (12-38)	41	11	27 (15-43)	-4 (-22-14) 0.663	NA	

For peer review only - http://bmjopen.bmj.com/site/about/guidelines.xhtml

* P-value based on t-test. ** Test for linear trend based on likelihood ratio test. *** P-value for interaction between area (intervention) and survey year (2019). Models of household survey indicators adjusted for maternal education. Denominators vary due to missing data.

For peer review only

Change in coverage 2016-19 by household wealth quintile

Six indicators from the household survey data demonstrated population level coverage improvement over time and were included in the equity analysis (Figure 3). Five of these were inequitable at baseline, with lower coverage among the poorest households. By 2019, although the change over time in coverage was even across all quintiles, baseline patterns of inequity persisted for three indicators: women attending at least four ANC visits (p=0.095), administration of uterotonics (p=0.118), and delayed bathing of the newborn (p=0.671). The improvement over time was inequitable for two indicators, with women in the poorest households benefiting the least: clean cord care (p=0.020) and facility birth (p=0.005). By contrast, improvement over time for at least one ANC was pro-poor (p=0.055), with coverage improving by 17 percentage points among the poorest households (from 58% to 75%), and by five percentage points to 91% among women in the least-poor households.

For peer review only

For peer review only - http://bmjopen.bmj.com/site/about/guidelines.xhtml

DISCUSSION

Over a four-year period in this rural and resource-constrained setting, this government-led maternal and newborn health partnership was able to improve several high impact indicators over and above any background changes observed in comparison areas. However, there was a marked lack of improvement in timely post-natal or post-partum care, coverage of which remained below fifteen percent. Additionally, despite improvements in coverage indicators, socio-economic inequalities in the use of maternal and newborn health services generally persisted.

Multiple improvement efforts were implemented simultaneously by the partnership. For example, increases in ANC visits and facility births were supported by more than one implementing partner. This reinforcement enabled the utilization coverage for these services to improve, even when historical data demonstrated little change during previous initiatives (41, 42). Changes to quality of care were mixed. We saw substantial increases in input quality where implementing partner actions directly strengthened pre-existing systems, for example where NGOs strengthened and managed existing supply chains for medicines and commodities and returned management to the government in a phased manner. However, this success was not replicated with process quality indicators. There was no excess improvement in process indicators of quality ANC, such as measuring blood pressure and taking a urine and blood sample during an ANC visit. Staffing and workload were especially challenging and it was not uncommon for one staff member to be attending to multiple women and babies (43). The challenge of changing health worker behaviour is well-established in the literature (44, 45). Challenges in providing complete and timely care have been noted in other low and middle income settings (46).

Coverage of timely post-natal and post-partum care for both home and facility births remained persistently low. This challenge is not unique to Gombe State, with very low coverage shown in other states of Nigeria (25), and the sub-Saharan African region more widely (47). Commonly reported barriers were present, for example sub-optimal demand generation, inadequate staffing and workload challenges, and multiple socio-cultural barriers (45, 46). However, the power of the partnership to address other apparently intractable problems was not harnessed in relation to timely post-natal and post-partum care visits, and actors did not conceive successful mechanisms that could address the problem in their setting. The role of community-based ward development committees, whose members include fathers, husbands and community leaders, had an important role in demand-generation, advocacy and accountability in this setting. NGO partners aimed to leverage the power and influence of ward committees for postnatal health, but these were enacted towards the end of our study period. Nonetheless, we suggest that such engagement is important for shifting norms and expectations where cultural barriers may restrict new mothers from returning to facilities soon after birth or accepting non-familial visitors during the immediate post-partum period (48, 49).

The Gombe Partnership for Maternal and Newborn Health achieved greater success than other recent NGO driven attempts and this study suggests the importance of the nature of the partnership and the mechanisms of implementation. The partnership was government-led with priorities and implementation guided by policy priorities (50, 51). This engendered political will. Further, good leadership, governance and accountability facilitated successful implementation despite a change in government during the 2019 general elections, which fell six months before the final data collection period of this study. The importance of political engagement and leadership has been highlighted by many, including the extensive work of Gilson and colleagues (52). From this foundation of cooperation, the intervention components and implementation plan were built to achieve a

For peer review only - http://bmjopen.bmj.com/site/about/guidelines.xhtml

common goal. From the inception, a plan for sustainability and transition from NGO implementation and donor-financing was in place (24). A substantial focus on community participation and accountability was also included, principally through the role of ward development committees.

Implementation was pragmatic and a strong emphasis was placed on adoption into the health system (20). The government and partners met six monthly to examine monitoring and evaluation data, assess progress, highlight challenges and plan for improvements. A recent systematic review has reported that many of these approaches, including a process of data-based feedback and adaptation have been important in the vertical scale-up and institutionalisation of public health interventions (53).

Strengths and limitations

This was a large and adequately-powered study which drew on multiple data sources and time points, and consistently applied globally recommended measurement standards. However, there were three noteworthy limitations. First, as for all maternal and newborn health data collected through maternal self-report, there was the possibility of recall bias, despite our relatively short twelve-month recall period. Further, we cannot discount the possibility that some women were not able to accurately report on all questions (54). Nonetheless, we would not expect recall bias or poor validity to be differential between groups or between surveys. Second, the packaging of interventions means that successful outcomes cannot be attributed to a single intervention, rather we can only conclude that the interventions in this health system strengthening package collectively contributed to the effects observed. Third, selection of intervention areas by government was pragmatic and baseline characteristics between areas were not balanced. Despite adjustment in multi-variate models and an analysis strategy that adjusted for baseline starting points, some residual confounding by area may remain, potentially overestimating effects of the intervention. The selection of intervention areas and the inability to build in buffer zones also resulted at times in close geographic boundaries between comparison and intervention areas. This may have enabled spill-over of effects created by women seeking care and utilising services from a facility that did not correspond to the intervention status of the ward in which she lived. This could have underestimated effects.

CONCLUSIONS

After four years, this government-led programme achieved improvements in life-saving interventions for mothers and newborns, demonstrating that even in settings where resources are constrained, the needle can be shifted in the right direction. In this example, multiple interventions that spanned different health system pillars were implemented together as a package and it is plausible that addressing demand and supply problems simultaneously was important. But we conclude that essential and reproducible elements of this programme lie not for the most part in the individual components of the intervention, but in the way the programme was designed and implemented with its focus on government leadership and strong stakeholder partnership.

For peer review only

For peer review only - http://bmjopen.bmj.com/site/about/guidelines.xhtml

Figure Legends

Figure 1: Intervention components by Health System Strengthening Building Block

Abbreviations: Health System strengthening (HSS), Primary Health Centre (PHC), Community Health Extension Worker (CHEW), Village Health Worker (VHW), Health Management Information System (HMIS), Maternal Perinatal Death Surveillance and Response (MPDSR), Maternal and Newborn Health (MNH).

Figure 1: Study timeline

Abbreviations: Maternal and Newborn Health (MNH)

Figure 2: Equiplot of change in coverage 2016-19 by household wealth quintile

Box 1: Indicator definitions

Note: data were collected for women aged 13–49 years. However, no women <15 years of age had a live birth in the 12 months before the survey.

Abbreviations: Antenatal care (ANC).

For peer review only

For peer review only - http://bmjopen.bmj.com/site/about/guidelines.xhtml

Acknowledgements

We are grateful to the women and families who participated and shared their experiences. We would like to thank all the health facility participants and birth attendants who took the time to contribute to our study. We would like to acknowledge Inuwa Jalingo from Data Research and Mapping Consult and recognise the contribution of Data Research and Mapping Consult field staff and supervisors who conducted the surveys. We would like to acknowledge the contribution of Vincent Ahonsi, Ester Agboa, and Tunde Segun from Evidence for Action, as well as Wale Adeleye from Champions for Change. Sincere thanks to all collaborators at the Gombe State Primary Health Care Development Agency, Gombe State Ministry of Health, Society for Family Health Nigeria, Pact Nigeria, Champions for Change, and Evidence for Action Mamaye. We would also like to acknowledge the contribution of our respected colleague and team member, Deepthi Wickremasinghe from the London School of Hygiene and Tropical Medicine, who sadly died on 2nd April 2020. Her wisdom, kindness and dedication to improving maternal and newborn health are greatly missed.

Contributions

BW conducted the analysis and drafted the manuscript. EB and EA provided statistical advice. BW, EB and JE verified the underlying data. NU, EB, AB, JE, KM, JS, NS and TM all provided important intellectual input. JA, ABB, MO, RS, AMG and AS coordinated study implementation and contributed to country data interpretation. UAU coordinated data collection activities and contributed to country data interpretation. TM and JS conceived the study. All authors have read, critically reviewed the manuscript and approved the final version of the manuscript.

Competing interests

All authors have completed the Unified Competing Interest form and declare no conflicts of interest.

Funding

The research was supported by IDEAS - Informed Decisions for Actions to improve maternal and newborn health (http://ideas.lshtm.ac.uk), which is funded through a grant from the Bill & Melinda Gates Foundation (Gates Global Health Grant Number: INV-007644) to the London School of Hygiene & Tropical Medicine.

Disclaimer

The funder of this study had no role in the study's design or conduct, data collection, analysis or interpretation of results, writing of the paper, or decision to submit for publication. The corresponding author had access to all study data and responsibility for the decision to submit the paper for publication.

Data sharing statement

De-identified participant datasets, protocols and questionnaires are uploaded to the LSHTM Data Repository. Data are available on reasonable request from the principal investigator of the IDEAS project and co-author for this manuscript, TM (ORCID id 0000-0002-4228-4334). Reuse permitted on request.

For peer review only

For peer review only - http://bmjopen.bmj.com/site/about/guidelines.xhtml

REFERENCES

1. Akseer N, Lawn JE, Keenan W, Konstantopoulos A, Cooper P, Ismail Z, et al. Ending preventable newborn deaths in a generation. International journal of gynaecology and obstetrics: the official organ of the International Federation of Gynaecology and Obstetrics. 2015;131 Suppl 1:S43-8.

2. GBD 2015 Maternal Mortality Collaborators. Global, regional, and national levels of maternal mortality, 1990-2015: a systematic analysis for the Global Burden of Disease Study 2015. Lancet (London, England). 2016;388(10053):1775-812.

3. PMNCH. A Global Review of the Key Interventions Related to Reproductive, Maternal, Newborn and Child Health (Rmnch). Geneva, Switzerland: The Partnership for Maternal, Newborn & Child Health. 2011.

4. Ronsmans C, Graham WJ. Maternal mortality: who, when, where, and why. Lancet (London, England). 2006;368(9542):1189-200.

5. WHO. Every newborn: an action plan to end preventable deaths. World Health Organization: Geneva. 2014.

6. WHO. Strategies towards ending preventable maternal mortality (EPMM). World Health Organization: Geneva. 2015.

7. Deorari A, Koganti RA. Closing the practice-to-outcome gap: lessons from the BetterBirth study. The Lancet Global health. 2019;7(8):e992-e3.

8. Gabrysch S, Nesbitt RC, Schoeps A, Hurt L, Soremekun S, Edmond K, et al. Does facility birth reduce maternal and perinatal mortality in Brong Ahafo, Ghana? A secondary analysis using data on 119 244 pregnancies from two cluster-randomised controlled trials. The Lancet Global health. 2019;7(8):e1074-e87.

9. Marx Delaney M, Miller KA, Bobanski L, Singh S, Kumar V, Karlage A, et al. Unpacking the null: a post-hoc analysis of a cluster-randomised controlled trial of the WHO Safe Childbirth Checklist in Uttar Pradesh, India (BetterBirth). The Lancet Global health. 2019;7(8):e1088-e96.

10. Alkema L, Chou D, Hogan D, Zhang S, Moller AB, Gemmill A, et al. Global, regional, and national levels and trends in maternal mortality between 1990 and 2015, with scenario-based projections to 2030: a systematic analysis by the UN Maternal Mortality Estimation Inter-Agency Group. Lancet (London, England). 2016;387(10017):462-74.

11. WHO. Trends in maternal mortality 2000 to 2017: estimates by WHO, UNICEF, UNFPA, World Bank Group and the United Nations Population Division. World Health Organization: Geneva. 2019.

12. Countdown to 2030 Collaboration. Countdown to 2030: tracking progress towards universal coverage for reproductive, maternal, newborn, and child health. Lancet (London, England). 2018;391(10129):1538-48.

13. UNICEF, WHO, World Bank, UN-DESA Population Division. Levels and trends in child mortality report 2019 Estimates developed by the UN Inter-agency Group for Child Mortality Estimation. United Nations Children's Fund: New York. 2019.

14. Amouzou A, Leslie HH, Ram M, Fox M, Jiwani SS, Requejo J, et al. Advances in the measurement of coverage for RMNCH and nutrition: from contact to effective coverage. BMJ global health. 2019;4(Suppl 4):e001297.

15. Carvajal-Aguirre L, Amouzou A, Mehra V, Ziqi M, Zaka N, Newby H. Gap between contact and content in maternal and newborn care: An analysis of data from 20 countries in sub-Saharan Africa. Journal of global health. 2017;7(2):020501.

16. Hodgins S, D'Agostino A. The quality-coverage gap in antenatal care: toward better measurement of effective coverage. Global health, science and practice. 2014;2(2):173-81.

17. Kruk ME, Gage AD, Arsenault C, Jordan K, Leslie HH, Roder-DeWan S, et al. High-quality health systems in the Sustainable Development Goals era: time for a revolution. The Lancet Global Health. 2018.

For peer review only - http://bmjopen.bmj.com/site/about/guidelines.xhtml

18. Kruk ME, Gage AD, Joseph NT, Danaei G, Garcia-Saiso S, Salomon JA. Mortality due to low-quality health systems in the universal health coverage era: a systematic analysis of amenable deaths in 137 countries. Lancet (London, England). 2018;392(10160):2203-12.
19. Ng M, Fullman N, Dieleman JL, Flaxman AD, Murray CJ, Lim SS. Effective coverage: a metric for monitoring Universal Health Coverage. PLoS Med. 2014;11(9):e1001730.
20. Yamey G. What are the barriers to scaling up health interventions in low and middle income countries? A qualitative study of academic leaders in implementation science. Globalization and health. 2012;8:11.
21. Wehrmeister FC, Fayé CM, da Silva ICM, Amouzou A, Ferreira LZ, Jiwani SS, et al. Wealth-related inequalities in the coverage of reproductive, maternal, newborn and child health interventions in 36 countries in the African Region. Bulletin of the World Health Organization. 2020;98(6):394-405.
22. Barros AJD, Wehrmeister FC, Ferreira LZ, Vidaletti LP, Hosseinpoor AR, Victora CG. Are the poorest poor being left behind? Estimating global inequalities in reproductive, maternal, newborn and child health. BMJ global health. 2020;5(1):e002229.
23. Gilson L, Lehmann U, Schneider H. Practicing governance towards equity in health systems: LMIC perspectives and experience. International journal for equity in health. 2017;16(1):171.
24. Spicer N, Hamza YA, Berhanu D, Gautham M, Schellenberg J, Tadesse F, et al. 'The development sector is a graveyard of pilot projects!' Six critical actions for externally funded implementers to foster scale-up of maternal and newborn health innovations in low and middle-income countries. Globalization and health. 2018;14(1):74.
25. National Population Commission and ICF. Nigeria Demographic and Health Survey 2018. Key Indicators Report. Abuja, Nigeria, and Rockville, Maryland, USA; 2019.
26. Izugbara CO, Wekesah FM, Adedini SA. Maternal Health in Nigeria. A Situation Update. Nairobi, Kenya: African Population and Health Research Centre; 2016.
27. National Bureau of Statistics (NBS) and United Nations Children's Fund (UNICEF). Multiple Indicator Cluster Survey 2016-17, Survey Findings Report. Abuja, Nigeria: National Bureau of Statistics and United Nations Children's Fund. 2017.
28. Bhattacharya AA, Umar N, Audu A, Felix H, Allen E, Schellenberg JRM, et al. Quality of routine facility data for monitoring priority maternal and newborn indicators in DHIS2: A case study from Gombe State, Nigeria. PloS one. 2019;14(1):e0211265.
29. Nigeria Health Management Information System, version 2013: Gombe State facility deliveries, by facility type, January-December 2018 [Internet]. Nigeria District Health Information System, version 2. 2019.
30. Gombe State Government. History of Gombe State, Nigeria. online: http://gombestategovng/history-2/ [accessed on 9 September 2019]. 2018.
31. Gombe State Ministry of Health. GOMBE state government strategic health development plan 2010-2015. online: https://drivegooglecom/file/d/0B1DAmtM1BcbMeVBRUjNEakViXzQ/view [accessed on 19 August 2020]. 2010.
32. WHO. Monitoring the Building Blocks of Health Systems: a handbook of indicators and their measurement strategies. World Health Organization: Geneva. 2010.
33. Wickremasinghe D AHY, Umar N, Willey B, Okolo M, Gana A, Shuaibu A, Anyanti J, Marchant T, Spicer N. 'A seamless transition': how to sustain a community health worker scheme within the health system of Gombe state, northeast Nigeria. Health Policy and Planning. 2021.
34. PACT. Pact mothers group becomes a leader in community development 2019 [Available from: https://www.pactworld.org/features/pact-mothers-group-becomes-leader-community-development.
35. MAMAYE, Babaranti M. The MamaYe accountability mechanism in Gombe will ensure that policy makers fulfil their promises. 2015 [Available from: https://mamaye.org/blog/gombe-assembly-sets-priority-health-sector.

For peer review only - http://bmjopen.bmj.com/site/about/guidelines.xhtml

36. IDEAS. The Pathway to Improved Maternal and Newborn Health Outcomes Use of data for maternal and newborn health in Gombe State, Nigeria. 2016.
37. Habicht JP, Victora CG, Vaughan JP. Evaluation designs for adequacy, plausibility and probability of public health programme performance and impact. International journal of epidemiology. 1999;28(1):10-8.
38. The DHS Program. DHS Model Questionnaires. online: https://wwwdhsprogramcom/What-We-Do/Survey-Types/DHS-Questionnairescfm [accessed on 19 August 2020]
39. Health Statistics and Information Systems, WHO. Service Availability and Readiness Assessment (SARA): an annual monitoring system for service delivery. Reference Manual, Version 2.2. World Health Organization: Geneva. 2015.
40. Kruk ME, Leslie HH, Verguet S, Mbaruku GM, Adanu RMK, Langer A. Quality of basic maternal care functions in health facilities of five African countries: an analysis of national health system surveys. The Lancet Global health. 2016;4(11):e845-e55.
41. Marchant T, Beaumont E, Makowiecka K, Berhanu D, Tessema T, Gautham M, et al. Coverage and equity of maternal and newborn health care in rural Nigeria, Ethiopia and India. CMAJ : Canadian Medical Association journal = journal de l'Association medicale canadienne. 2019;191(43):E1179-e88.
42. Tomlin K, Berhanu D, Gautham M, Umar N, Schellenberg J, Wickremasinghe D, et al. Assessing capacity of health facilities to provide routine maternal and newborn care in low-income settings: what proportions are ready to provide good-quality care, and what proportions of women receive it? BMC Pregnancy Childbirth. 2020;20(1):289.
43. Exley J, Hanson C, Umar N, Willey B, Shuaibu A, Marchant T. The provision of essential evidence-based interventions during facility-based childbirth: cross sectional observations of births in northeast Nigeria. BMJ Open. forthcoming.
44. Marx Delaney M, Maji P, Kalita T, Kara N, Rana D, Kumar K, et al. Improving Adherence to Essential Birth Practices Using the WHO Safe Childbirth Checklist With Peer Coaching: Experience From 60 Public Health Facilities in Uttar Pradesh, India. Global health, science and practice. 2017;5(2):217-31.
45. Rowe AK, Rowe SY, Peters DH, Holloway KA, Chalker J, Ross-Degnan D. Effectiveness of strategies to improve health-care provider practices in low-income and middle-income countries: a systematic review. The Lancet Global health. 2018;6(11):e1163-e75.
46. Munabi-Babigumira S, Glenton C, Lewin S, Fretheim A, Nabudere H. Factors that influence the provision of intrapartum and postnatal care by skilled birth attendants in low- and middle-income countries: a qualitative evidence synthesis. The Cochrane database of systematic reviews. 2017;11(11):Cd011558.
47. The African Academy of Science, The Academy of Medical Science. From minding the gap to closing the gap: Science to transform maternal and newborn survival and stillbirths in sub-Saharan Africa in the Sustainable Development Goals era. Workshop Report 4–5 September 2018 Nairobi, Kenya. 2019.
48. Amare Y, Scheelbeek P, Schellenberg J, Berhanu D, Hill Z. Early postnatal home visits: a qualitative study of barriers and facilitators to achieving high coverage. BMC public health. 2018;18(1):1074.
49. Hill Z, Scheelbeek P, Hamza Y, Amare Y, Schellenberg J. Are We Using the Right Approach to Change Newborn Care Practices in the Community? Qualitative Evidence From Ethiopia and Northern Nigeria. Global health, science and practice. 2020.
50. Federal Government of Nigeria. Second National Strategic Health Development Plan 2018-2022. online: http://wwwhealthgovng/doc/NSHDP%20II%20Finalpdf [accessed on 18 August 2020]. 2019.
51. Network for Improving Quality of Care for Maternal. Quality of Care in Nigeria. online: http://wwwqualityofcarenetworkorg/country-data/nigeria [accessed on 18 August 2020]. 2019.

For peer review only - http://bmjopen.bmj.com/site/about/guidelines.xhtml

52. Gilson L, Agyepong IA. Strengthening health system leadership for better governance: what does it take? Health Policy and Planning. 2018;33(suppl_2):ii1-ii4.
53. Bulthuis SE, Kok MC, Raven J, Dieleman MA. Factors influencing the scale-up of public health interventions in low- and middle-income countries: a qualitative systematic literature review. Health Policy Plan. 2020;35(2):219-34.
54. Bhattacharya AA AE, Umar N, Usman AU, Felix H, Audu A, Schellenberg JR, Marchant T. Monitoring childbirth care in primary health facilities: a validity study in Gombe State, northeastern Nigeria. Journal of global health. 2019;9(2).

For peer review only

For peer review only - http://bmjopen.bmj.com/site/about/guidelines.xhtml

WHO HSS building block	Service Delivery	Health Workforce	Information	Products and technologies	Financing	Leadership Governance
Implemented in intervention area only	Quality improvement teams in PHCs<br>VHW links with facilities<br>Forum of mothers in law<br>Forum of males<br>Mothers groups	Task shifting and training CHEWs<br>Training and deployment of VHWs<br>Financial incentives for VHWs		Enhancing supply chains to PHCs		Strengthening Ward Development Committees<br>Organizational development
Implemented state-wide	Emergency transport Scheme		HMIS strengthening<br>MPDSR information package and support to committee<br>Score cards<br>Mass media events		Leveraging the State Level Accountability Mechanism to advocate for appropriate budget release for MNH	

For peer review only

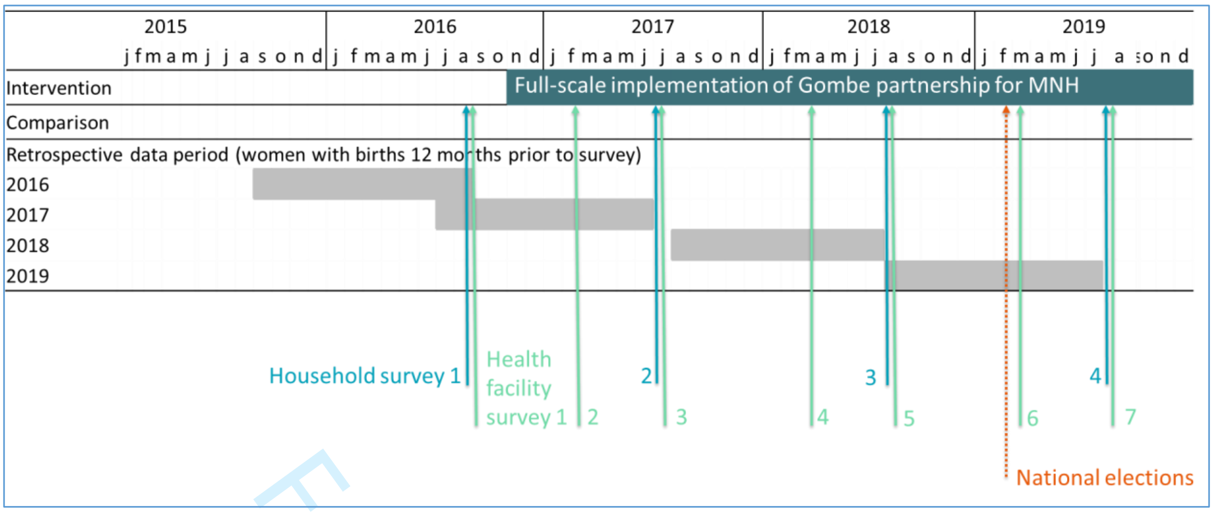

For peer review only

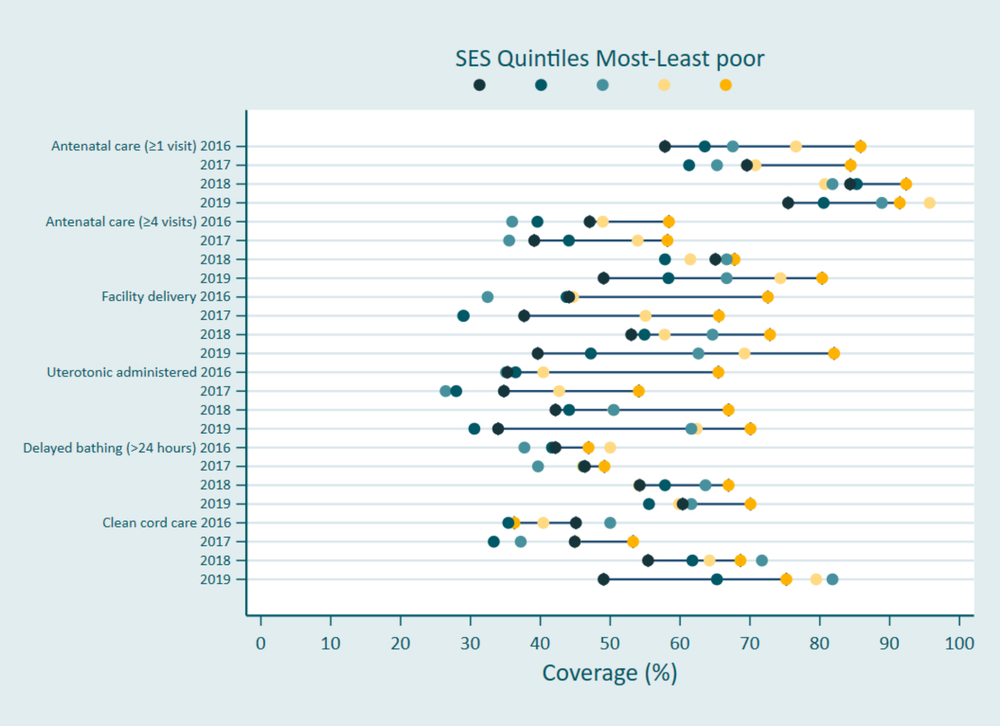

Figure 3: Equiplot of change in coverage 2016-19 by household wealth quintile

372x270mm (72 x 72 DPI)
